# Transcriptome characterization via 454 pyrosequencing of the annelid *Pristina leidyi*, an emerging model for studying the evolution of regeneration

**DOI:** 10.1186/1471-2164-13-287

**Published:** 2012-06-29

**Authors:** Kevin G Nyberg, Matthew A Conte, Jamie L Kostyun, Alison Forde, Alexandra E Bely

**Affiliations:** 1 Department of Biology, University of Maryland, College Park, MD, 20742, USA; 2 Department of Integrative Biology, University of Guelph, Guelph, ON, N1G 2W1, Canada; 3 Current address: Department of Biology, Indiana University, Bloomington, IN, 47401, USA

**Keywords:** Transcriptome, 454 pyrosequencing, Regeneration, Evolution, Asexual reproduction, Fission, Annelid, *Pristina leidyi*, Cell signaling, Wnt signaling

## Abstract

**Background:**

The naid annelids contain a number of species that vary in their ability to regenerate lost body parts, making them excellent candidates for evolution of regeneration studies. However, scant sequence data exists to facilitate such studies. We constructed a cDNA library from the naid *Pristina leidyi*, a species that is highly regenerative and also reproduces asexually by fission, using material from a range of regeneration and fission stages for our library. We then sequenced the transcriptome of *P. leidyi* using 454 technology.

**Results:**

454 sequencing produced 1,550,174 reads with an average read length of 376 nucleotides. Assembly of 454 sequence reads resulted in 64,522 isogroups and 46,679 singletons for a total of 111,201 unigenes in this transcriptome. We estimate that over 95% of the transcripts in our library are present in our transcriptome. 17.7% of isogroups had significant BLAST hits to the UniProt database and these include putative homologs of a number of genes relevant to regeneration research. Although many sequences are incomplete, the mean sequence length of transcripts (isotigs) is 707 nucleotides. Thus, many sequences are large enough to be immediately useful for downstream applications such as gene expression analyses. Using in situ hybridization, we show that two Wnt/β-catenin pathway genes (homologs of *frizzled* and *β-catenin*) present in our transcriptome are expressed in the regeneration blastema of *P. leidyi*, demonstrating the usefulness of this resource for regeneration research.

**Conclusions:**

454 sequencing is a rapid and efficient approach for identifying large numbers of genes in an organism that lacks a sequenced genome. This transcriptome dataset will be a valuable resource for molecular analyses of regeneration in *P. leidyi* and will serve as a starting point for comparisons to non-regenerating naids. It also contributes significantly to the still limited genomic resources available for annelids and lophotrochozoans more generally.

## Background

The process of regeneration, or the replacement of lost body parts, has long captured the interest of biologists. While early experiments on crayfish [1] and *Hydra*[2] demonstrated the remarkable abilities of some animals to develop lost parts anew, it is also clear that many animals, including humans, do not possess such abilities. The ability to regenerate is thought to have been lost over the course of evolution in many animal lineages [3-5]. Despite recent advances in knowledge of the molecular and developmental basis of regeneration in a variety of animal systems [6-8], little is currently known about the developmental and evolutionary mechanisms that drive loss of regeneration ability [5]. Understanding this phenomenon requires a comparative approach and the identification and development of animal systems that show variation in regeneration ability among closely related species.

The naid annelids are among a small number of documented groups in which regeneration ability varies among close relatives [5,9-14], making them a good model for studying the loss of regeneration. Naids (the minimal clade including both the Naidinae and Pristininae) are a group of small aquatic oligochaete worms, many of which can reproduce asexually by fission [15]. Many naids, including *Pristina leidyi*, possess excellent regeneration abilities, being able to regrow both their heads and tails after amputation. Following amputation, tissues at the wound site actively proliferate and form a regeneration blastema (a mass of undifferentiated cells) which ultimately differentiates to give rise to regenerated structures [16]. The ability to regenerate anteriorly and posteriorly is thought to be ancestral for the clade. However, recent experiments indicate that head regeneration ability has been lost at least three times within the naids, allowing multiple independent comparisons between regenerating and non-regenerating species [9,10,17]. The degree of loss of the regeneration machinery can vary between lineages, suggesting that different developmental mechanisms may underlie independent evolutionary losses of regeneration [10]. Thus, in the naids, evolution has crafted an ideal experiment for investigating loss of regeneration.

Much recent work on the developmental basis of regeneration has focused on the role of signaling pathways, such as the Wnt pathway, in recruiting stem cells and promoting morphogenesis in regenerated tissues [18-29]. In order to investigate the role of signaling pathways and other molecules in variation of regeneration ability, genomic resources are needed for the naids. Recent advances in high-throughput sequencing and bioinformatic analyses have made transcriptome sequencing feasible for discovering novel genes in non-model systems.

454 pyrosequencing, with sequence reads now approaching the length of traditional Sanger sequences, is ideal for transcriptome sequencing in a model that lacks a sequenced genome [30,31]. While the sequencing depth of 454 is modest compared to that of other deep sequencing technologies, 454 does offer depth orders of magnitude above what can be obtained via Sanger sequencing [32]. In addition, recent versions of the Newbler assembler from 454 allow for assembling sequences from cDNA, grouping presumptive gene isoforms together. Here, we describe the sequencing and assembly of a full run of 454 GS FLX sequencing with Titanium reagents from the regenerating annelid *P. leidyi*.

## Results and discussion

### Genome size estimation

We estimated the genome size of *P. leidyi* and four other naid species currently used in comparative regeneration studies [10]. Using Feulgen densitometry analysis, we estimated a C-value of 1.37 pg for *P. leidyi* and C-values ranging from 0.54 to 1.09 pg for the four other naid species (Additional file 1). Previously published estimates for two naid species are 1.53 and 3.23 pg, and the mean of values reported for oligochaetes is ~1.6 pg (range: 0.43 to 7.64) [33,34]. Thus, the genome size of *P. leidyi* is typical for this group.

### Construction of a partially normalized cDNA library

**Figure 1:**
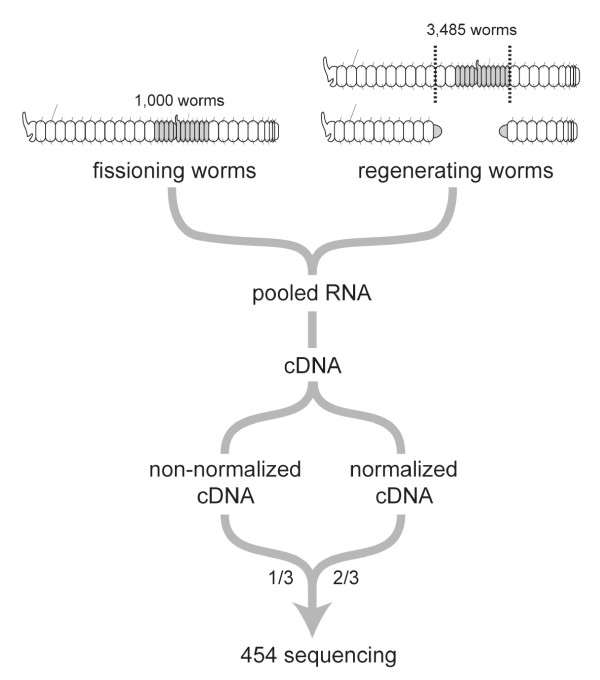
**Workflow of cDNA library construction.** A mixed-stage regeneration/fission cDNA library was generated from ~4,500 *P. leidyi* worms. Anteriorly and posteriorly regenerating worms were collected from 0 to 3.5 days after amputation (dotted lines mark amputation planes; gray terminal masses represent regeneration blastemas) and actively fissioning worms were also collected (gray shading marks intercalated head and tail tissue that forms during fission). Following RNA extraction and cDNA synthesis, a portion of the pooled cDNA was normalized. The final library sent for 454 sequencing consisted of 2/3 normalized and 1/3 non-normalized cDNA.

In order to maximize the discovery of genes in *P. leidyi*, we constructed a partially normalized cDNA library from mixed-stage regenerating and fissioning material (Figure 1). Regeneration and fission are highly similar processes that are thought to be evolutionarily related in these animals, with fission hypothesized to have evolved by co-option of regeneration [16,35]. Although the two processes are developmentally very similar, several studies have also demonstrated clear differences between the two [16,35]. We thus chose to include material from both regeneration and fission for this study to facilitate future studies of both processes. Furthermore, because *P. leidyi* worms fission continuously when well fed, we wanted to include fission material in this transcriptome as it represents a "baseline" process in these animals.

RNA was extracted from whole worms at multiple time points between the initiation of regeneration and its completion and from unamputated worms that were actively undergoing fission. cDNA was synthesized with an oligo-dT primer and a MINT full-length reverse-transcription kit. PCR assays indicated that the dried Spirulina powder used as food was not metabolically active and could not be detected by RT-PCR in the cDNA sample (Additional file 2).

**Figure 2:**
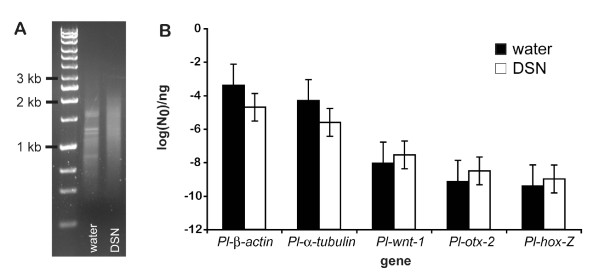
**Effectiveness of normalization of the cDNA library using duplex-specific nuclease.** (**A**) Agarose gel smears show that non-normalized cDNA (treated with water) has distinct bands representing highly expressed genes, but these bands are absent in the normalized sample treated with duplex-specific nuclease (DSN). (**B**) RT-PCR analysis of transcript levels indicates that representation of the highly expressed genes *Pl-β-actin* and *Pl-α-tubulin* in the library is decreased after normalization with DSN. Representation of three lowly expressed genes, *Pl-wnt-1*, *Pl-otx-2*, and *Pl-hox-Z*, is increased, consistent with successful normalization. Standard error bars are shown.

A portion of the cDNA library was subjected to normalization using a duplex-specific nuclease (DSN) in order to avoid repetitive sequencing of highly expressed genes [36,37]. Normalization efficiency was assayed using agarose gel smears and qPCR of select highly and lowly expressed genes (Figure 2). Highly expressed genes from non-normalized cDNA, visible as distinct bands on the agarose gel, were absent or greatly reduced in the normalized cDNA sample (Figure 2A). Furthermore, levels of select genes known to have high (*Pl-β-actin* and *Pl-α-tubulin*) or low (*Pl-wnt-1, Pl-otx-2*, and *Pl-hox-Z*) expression during *P. leidyi* regeneration, as previously determined by RT-PCR, were compared between normalized and non-normalized cDNA samples (Figure 2B). The two highly expressed genes, *Pl-β-actin* and *Pl-α-tubulin*, showed a reduction in transcript levels of over an order of magnitude upon normalization, while the proportional representation of the three lowly expressed genes increased in the library after normalization. Taken together, these data indicate successful normalization of the cDNA library.

The overall amount of cDNA is greatly reduced during normalization, making PCR amplification necessary to produce a sufficient quantity of cDNA for 454 sequencing. Because PCR has its own biases, particularly against large amplicons, we pooled an unamplified, non-normalized sample with a normalized sample in a 1:2 ratio to increase the representation of longer transcripts (Figure 1). This pooled cDNA library was used for 454 pyrosequencing.

### 454 pyrosequencing and transcriptome assembly

The combined cDNA library was sequenced using a 454 GS FLX sequencer with Titanium reagents, producing 1,550,174 sequence reads with an average length of 376 nt. Total sequence output was 583,020,992 nt (Table 1). The reads from this sequencing effort, collectively referred to as Pristina454RF (RF = Regeneration/Fission), have been deposited in the NCBI’s Short Read Archive (SRA) database [38] under accession # SRX110479.

**Table 1:** Sequence and assembly output

**454 sequencing results**			
# reads	1,550,174	mean read length	376 nt
# nucleotides	583,020,992		
**Newbler Assembler v2.3 assembly results**			
# contigs	186,015		
# isotigs	95,644	mean isotig length	707 nt
		mean max isotig length	549 nt
# isogroups	64,522		
# singletons	46,679		
# unigenes	111,201		
fraction captured transcripts		0.9699	
gene discovery rate		33.21 reads/new gene	

454 sequence reads were assembled using the Newbler Assembler v2.3 [31]. Sequence output using the cDNA option of Newbler v2.3 differs from that of traditional genomic assemblers (e.g. SeqMan NGen 2.0, CAP3, Newbler 2.2) by taking into account the possibility that multiple isoforms (e.g. alternative splice variants) of a gene may be present. Overlapping sequence reads are assembled into contigs, much like traditional assemblers. However, if multiple isoforms are present, a sequence read may contain a portion that aligns perfectly with the previously constructed contig and a portion that does not (with the point of divergence being, for example, an exon-exon junction). When this occurs, Newbler v2.3 breaks up the aligned sequences into multiple contigs. Sequences shared between multiple isoforms are retained as unique contigs, and any adjacent variant sequences are split off as their own unique contigs. Thus, a single gene isoform might be assembled into multiple contigs, and the same contig might be shared across multiple isoforms. Each putative isoform identified by Newbler v2.3 is termed an isotig, and the multiple isoforms for each gene are organized into isogroups, representing putative gene loci.

**Figure 3:**
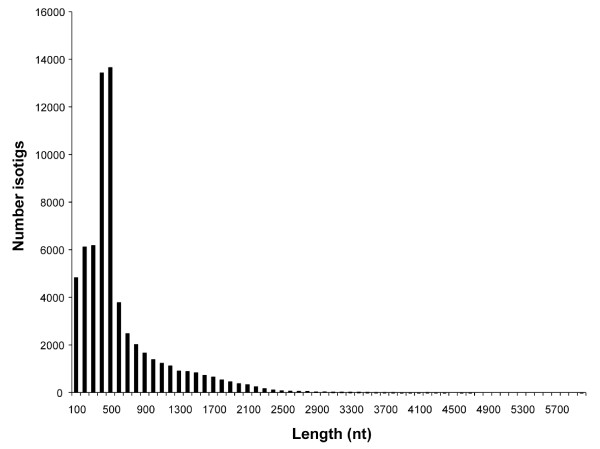
**Size distribution of largest isotig from each isogroup.** A size distribution of the largest isotig from each isogroup shows that most isotigs are several hundred nucleotides in length, though some isotigs are as large as several thousand nucleotides.

Newbler v2.3 identified 95,644 unique isotigs, with a mean length of 707 nt (Table 1), comprised of 186,015 unique contigs. Newbler grouped these into 64,522 unique isogroups (putative gene loci), and the mean of the largest isotig from each isogroup is 549 nt (Table 1, Figure 3). 46,479 of the original sequence reads could not be assembled with any other sequence and remained as singletons. In total, 111,201 unigenes (# isogroups + # singletons) were predicted by Newbler v2.3 (Table 1). Using the method of Susko and Roger (2004), we estimate that 96.99% of all genes contained within the cDNA sample are present in the 454 dataset (Table 1) [39,40]. At this level of sequencing, a new gene is expected to be discovered with every additional 33.21 sequence reads. The collection of isotigs and isogroups produced by this transcriptome assembly, referred to as Pristina454RF-N2.3, can be accessed directly at the BouillaBase EST Database [41]. General information about accessing and searching this transcriptome is provided at the Bely Lab Resources webpage [42].

### BLAST analysis of 454 isotigs

After assembly, the 95,644 isotigs were run through the EST2uni analysis pipeline in order to provide annotations from the UniProt database and create a searchable online BLAST interface [43]. The largest isotig from each isogroup was used as a representative for subsequent BLAST analyses (Tables 2,3). 17.7% of isogroups (11,388/64,522) had a significant BLAST hit (E-value < e^-10^) against the UniProt database (Table 2). The vast majority of these hits matched other animal sequences (96.1%), though the number matching lophotrochozoan taxa (the major bilaterian clade that includes annelids) was low (1.5%), presumably due to a dearth of lophotrochozoan sequences in UniProt itself (Table 2).

**Table 2:** Annotation of isotigs

**EST2uni BLAST results**		
queries	64,522 (max isotig per isogroup)	
BLAST hits	11,388 (17.7%, E-value < e^-10^)	
**Taxonomic identity of top UniProt hit**		
Eukaryotes	11,227	98.6%
Animals	10,945	96.1%
Deuterostomia	9,444	82.9%
Ecdysozoa	1,291	11.3%
Lophotrochozoa	174	1.5%
Fungi	110	1.0%
Plants	84	0.7%
Protists	88	0.8%
Prokaryotes	154	1.4%
Viruses	7	0.1%
Total BLAST hits	11,388	100%

**Table 3:** Isotig matches to UniProt database

	**UniProt ref. size**				
	**<250 aa**	**251-500 aa**	**501-750 aa**	**>750 aa**	**Total**
**Max isotigs**					
**5’ + 3’**	31.7%	8.2%	1.4%	<0.1%	6.5%
**5’ only**	22.1%	16.6%	11.4%	8.1%	13.0%
**3’ only**	26.4%	23.5%	15.1%	7.2%	16.1%
**neither end**	19.8%	51.7%	72.1%	84.6%	64.3%
**Total**	1383	3384	2578	4156	11,501

BLAST results suggest that our efforts to minimize environmental contamination were successful. BLAST searches against the *P. leidyi* isotig dataset using either a cnidarian or human 16 S sequence [*Hydra magnipapillata*: GenBank:NC_011220|:307-2044; *Homo sapiens*: GenBank: FJ794693.1|:1673-3230] retrieve isotigs matching to *Pristina* 16S as the only hits with any reasonable significance (E-value < 0.1), suggesting no metazoan contamination. Furthermore, only a small number of isotigs matched prokaryotic genes (1.4%) (Table 2) and BLAST searches of the *P. leidyi* isotigs using bacterial 16S RNA from either the proteobacterium *Escherichia coli* [GenBank:4924485] or the cyanobacterium *Arthrospira platensis* (Spirulina) [GenBank:FJ798612.1] return a very limited number of isotigs (only nine, and the same nine, isotigs for both searches; E-values < e^-2^). Interestingly, four of these isotigs match 16S from bacterial genera known to be common endosymbionts in animal intestines (gammaproteobacteria *Edwardsiella*/*Xenorhabdus*/*Photorhabdus*; bacteroidetes *Paenicardinium*/*Cardinium*). Thus, some bacterial sequences present in the transcriptome may represent the endemic gut flora of *P. leidyi*.

To assess how well 5’ and 3’ ends were captured in our dataset, isotigs with significant BLAST hits were compared to their counterparts in UniProt (Table 3). The proportion of isotigs with captured ends (within 10 amino acids of the corresponding end of the UniProt sequence) varies with the length of the coding sequence in UniProt. Isotigs matching shorter UniProt sequences are more likely to be complete on both the 5’ and 3’ ends than isotigs matching longer UniProt sequences. 31.7% of isotigs matching UniProt sequences of less than 250 amino acids are complete on both ends, while only a single isotig matching UniProt sequences greater than 750 amino acids is complete. In total, 6.5% of isotigs can be considered complete, 13.0% have captured the 5’ end, 16.1% have captured the 3’ end, and 64.3% have no matches against either end. While we estimate that we have captured the vast majority of transcripts in the original cDNA library (Table 1) and that there is no strong bias towards either 5’ or 3’ ends, it is clear that most of our unigenes consist of only partial transcript sequences. Further sequencing, either in a high-throughput manner or on a targeted basis with genes of interest, will be necessary to fill in these gaps.

### Gene ontology analysis of 454 isotigs

**Figure 4:**
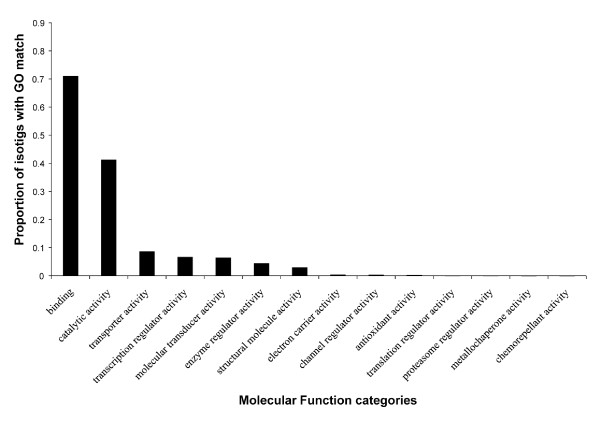
**Gene Ontology Biological Process designations of isotigs.** Representative isotigs were subjected to Gene Ontology (GO) analysis using Blast2GO. Categories are level 2 Biological Process designations. Proportion on the y-axis was calculated from the total number of representative isotigs that were annotated with GO terms (11,140).

The set of representative isotigs was also subjected to a Gene Ontology (GO) analysis using Blast2GO in order to determine whether genes with GO terms relevant to regeneration research could be identified [44,45]. 11,140 of the 64,522 representative isotigs were associated with GO terms. Significant numbers of these were associated with the Biological Process terms “developmental process” (27.5% of 11,140 GO-annotated isotigs searched), “signaling” (20.6%), “death” (7.3%), “cell proliferation” (6.6%), and “growth” (5.2%) (Figure 4). This analysis suggests that our dataset contains many genes that are likely to be involved in regeneration. Results for Molecular Function and Cellular Process GO searches are provided in Additional file 3.

### Identification of candidate regeneration genes

Using reciprocal BLAST searches between our transcriptome and publicly available sequences, we identified putative *P. leidyi* homologues of genes that have been implicated in regeneration in other regeneration models (Table 4). The genes listed here are active in a range of regeneration processes including wound healing, blastema formation, stem cell regulation, and controlling cell proliferation and morphogenesis [29,46,47]. Some genes were represented by multiple isogroups, likely indicating multiple unique homologs in *P. leidyi*. For example, there appear to be multiple homologs of *wnt* and *frizzled* in *P. leidyi*, which is consistent with what is known about these gene families in other annelids or lophotrochozoans more broadly [19,48,49].

**Table 4:** BLAST results for candidate regeneration genes

**Gene name**	**[References]**	**BLAST query**	**Isogroup #**
MMP	[50-52]	AY068367	00684, 55353
PIWI-like	[53-56]	DQ186986	08478
Nanos	[57,58]	EF153633	04448
β-catenin	[19,20,23]	EU296629	01340
Wnt	[18,24,25,27]	FJ463749	07867, 10773, 21383, 44961, 47307
Frizzled	[20,59]	AB201956	03233, 13291, 15038, 40749, 49382
GSK3β	[60]	DQ402057	03101, 07423, 11748, 64463
FGFR	[25,61,62]	NM_001090663	46357
JNK	[63,64]	NM_164900	01573
BMP	[65-67]	EF633689	05943, 26907
Noggin	[66-68]	EF633690	46857
Hh	[69-72]	NM_001088313	18046
Patched	[70-72]	AB504738	38195, 44428
Msx	[73,74]	AF061271	60588
Dlx	[75,76]	U59480	00856, 52001, 54688

### Independent confirmation of assembled transcripts

The utility of this transcriptome will ultimately be determined by whether these assembled sequences can be independently confirmed and manipulated for further studies. Because 454 sequencing has a nebulization step and is not performed using intact transcripts, Newbler v2.3 is unable to reconstruct with complete accuracy the actual gene isoforms present *in vivo*. Therefore, isotigs should be treated as predicted gene isoforms that require independent confirmation, such as by PCR assay. Contigs, on the other hand, already are well supported via the original sequencing and are expected to be true contiguous sequence and thus amplifiable by PCR.

**Figure 5:**
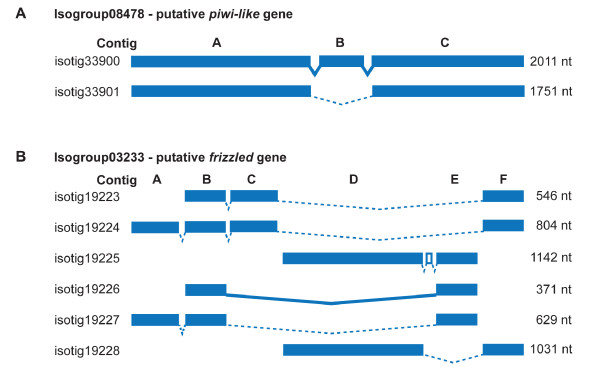
**Validation of transcript assembly for two isogroups.** Contigs and isotigs produced by the assembly are shown for two isogroups, (**A**) a putative *piwi-like* gene and (**B**) a putative *frizzled* gene. Blue boxes represent major contigs and V-shaped lines connect contigs that are adjacent to each other within the isotig. Short contigs of only a few nucleotides are omitted in this representation. Contigs that were independently confirmed by PCR and sequencing are represented as filled blue boxes (as opposed to open boxes) and connections between contigs that were independently confirmed by PCR and sequencing are indicated by solid V-shaped lines (as opposed to dotted lines). All major contigs and one isotig for each gene (isotigs 33900 (A) and 19226 (B)) were validated. Nucleotide sequence alignments are provided in Additional file 4.

We have used PCR assays to validate contigs and isotigs from our assembly for over 20 genes to date and discuss here results for two well-characterized isogroups as examples. One isogroup of interest (isogroup08478) was identified via BLAST as a member of the *piwi-like* gene family, which is implicated in stem cell regulation in several systems [53-55,77]. This isogroup consists of two isotigs, one comprised of three contigs and the other comprised of only two of the three contigs (Figure 5A, Additional file 4). We were able to recover by PCR and confirm by sequencing all three contigs and one of the isotigs for this isogroup. Another isogroup of interest (isogroup03233) was identified via BLAST as a *frizzled* gene, a major receptor in the Wnt signaling pathway [59]. The transcriptome assembly for this isogroup is more complex, as the isogroup consists of six isotigs and six contigs, with each isotig comprised of a different subset of contigs (Figure 5B, Additional file 4). Although the genomic order of some contigs remains unclear for this isogroup, all six contigs and one of the six isotigs were recovered by PCR and confirmed by sequencing. Thus, although some isotigs might be constructed as artifacts of the assembly process, PCR assays demonstrate that contigs and some isotigs can be independently validated. This indicates that this 454 transcriptome dataset will be highly valuable for further regeneration research.

**Figure 6:**
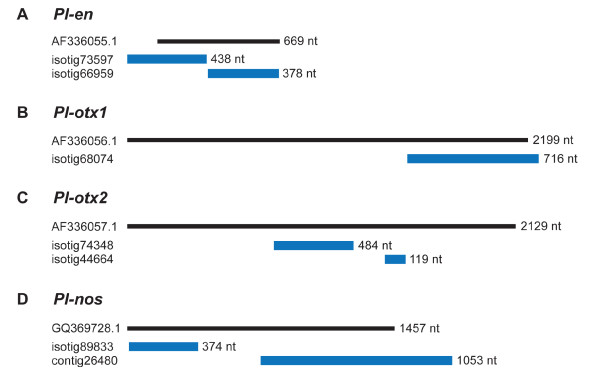
**Transcriptome coverage of four previously known gene sequences.** All four developmental genes previously sequenced from *P. leidyi* are represented in the transcriptome, although coverage is incomplete. Black bars represent previously known sequence for each gene (GenBank numbers on left) and blue bars represent transcriptome sequences matching to or extending the reference sequence (isotig/contig numbers on left).

Very limited sequence data were available for *P. leidyi* prior to the current sequencing effort, but it is worth noting that all four developmental genes that were previously isolated and characterized in this species [10,35] are present in this transcriptome. BLAST searches against the 454 dataset using the previously published gene sequences for *Pl-enPl-otx1Pl-otx2*, and *Pl-nos* as queries retrieved one isotig matching *Pl-otx1* and two isotigs matching each of the other three genes (Figure 6). Alignment of transcriptome sequences to published sequences provides validation for the transcriptome assembly for all four genes (Figure 6). However, for *Pl-enPl-otx2*, and *Pl-nos*, the two isotigs retrieved are non-overlapping, indicating the transcriptome sequences remain unresolved. For *Pl-en* and *Pl-nos*, the isotig or isotigs in the transcriptome cover most of the previously known sequence (and even extend the known sequence), but for *Pl-otx1* and *Pl-otx2* the transcriptome sequences represent only ~1/3 of the previously known sequence (Figure 6). Thus, although gene representation appears to be high in this transcriptome, we expect that further sequencing, either in a high-throughput manner or on an individual basis, will be necessary to determine full-length sequences of many transcripts.

### Expression of wnt/β-catenin pathway genes during regeneration

To further demonstrate the utility of our sequencing effort for regeneration studies, we examined the expression patterns of two genes present in the transcriptome, homologs of *frizzled* (*fz*) and *β-catenin* (*β-cat*). These genes were chosen because they are components of the Wnt/β-catenin pathway, an important cell signaling pathway implicated in numerous developmental processes, including regeneration [18-29]. We identified from our transcriptome several homologs of *fz* (a Wnt ligand receptor) and a single homolog of *β-cat* (a multifunctional protein that acts as a transcription factor when Wnt signaling is activated). We examined expression of one of these *fz* homologs, *Pl-fzA*, and the homolog of *β-catPl-β-cat*, by whole mount in situ hybridization of regenerating and fissioning *P. leidyi*.

**Figure 7:**
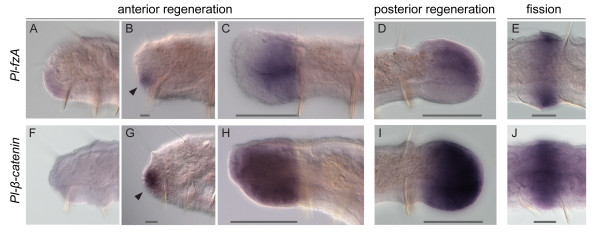
**Expression of two Wnt/β-catenin pathway genes during regeneration and fission.** Whole mount in situ hybridizations of *Pl-fzA* (**A**-**E**) and *Pl-β-cat* (**F**-**J**) show that both genes are expressed in the developing regeneration blastema as well as in new tissues forming by fission. (A-D) Following anterior amputation, *Pl-fzA* expression is not detectable (or only faintly so) before a blastema forms (A: 12 hours post amputation (hpa)), begins to be expressed in the early blastema (B: 1 day post amputation (dpa)) and persists through mid-stages of regeneration (C: 2 dpa). *Pl-fzA* is expressed in a similar fashion during posterior regeneration (D: 2 dpa). (F-I) Following anterior amputation, *Pl-β-cat* is similarly not detectable (or only faintly so) prior to blastema formation (F: 12 hpa), begins to be expressed in the early blastema (G: 1 dpa), and persists through mid-stages of regeneration (H: 3 dpa). *Pl-β-cat* is also expressed during posterior regeneration (I: 2 dpa). (E, J) During fission, both genes are expressed in developing fission zones (E, J: early fission - stage B), the transverse regions of tissue from which a new head and tail form (see Figure 1). All panels are lateral views with anterior to the left. Dark gray bars mark the extent of new tissue, i.e., the regeneration blastema or fission zone. Arrows point to early phase of expression of each gene on day 1.

During both anterior and posterior regeneration, *Pl-fzA* and *Pl-β-cat* are expressed strongly and specifically within the regeneration blastema, the mass of cells from which the new structures will develop (Figure 7: A-D, F-I). For both genes, expression becomes detectable at the wound site between 12 and 24 hours after amputation, around the time a blastema becomes visible, and expression then broadens as the blastema grows. Expression remains high through mid-stages of regeneration, gradually fading as the blastema differentiates. *Pl-fzA* is expressed diffusely in much of the blastema but is weak ventrally and highest in a lateral band on either side of the blastema at mid-stages of regeneration. *Pl-β-cat* shows broad and strong expression throughout the blastema. Consistent with the idea that fission and regeneration are evolutionarily related processes, both genes are also expressed in new tissue developing by fission, in patterns largely similar to those during regeneration (Figure 7: E, J). In situ hybridizations using control sense probes yield only light diffuse staining suggestive of probe trapping. Expression patterns of *Pl-fzA* and *Pl-β-cat* are distinct from each other and from those of other genes investigated in this species, further indicating specificity of our in situ results.

Our results provide the first expression data for *frizzled* or *β-catenin* genes during annelid regeneration and strongly implicate Wnt signaling in *P. leidyi* regeneration. They also add to the accumulating data showing a close developmental relationship between regeneration and fission in these animals. More broadly, these findings demonstrate that sequences from this transcriptome can provide new insights into annelid development, setting the stage for future comparative studies of annelid regeneration.

### Applications for further regeneration research

This transcriptome dataset provides a valuable new resource for regeneration research in annelids. The work described here already demonstrates the utility of this dataset: our GO analysis suggests that a large number of genes relevant for regeneration research are represented, our sequence confirmation assays show that putative transcripts from our assembly can be independently validated, and our expression studies demonstrate that genes expressed during regeneration are indeed present in this transcriptome and can provide new insight into annelid regeneration.

This transcriptome resource promises to accelerate regeneration research in *P. leidyi* and provides a stepping-stone to studies of regeneration failure in closely related, non-regenerating naid species. This dataset greatly facilitates gene discovery, allowing genes of interest to be quickly identified and characterized by RT-PCR or *in situ* hybridization. Importantly, this resource can also provide a reference transcriptome for larger, genome-scale studies, such as high-throughput analyses of gene expression by microarrays or RNA-Seq [78-80]. Extending these approaches to closely related regenerating and non-regenerating naid species holds great promise for elucidating the genetic basis of both regeneration success and failure.

## Conclusions

This transcriptome sequencing project has produced the first genomic-type sequence data for any of the naid annelids, a promising group for understanding regeneration loss. This dataset also represents the first regeneration-based, large-scale sequence database for annelids as a whole and thus provides a valuable resource for regeneration research more broadly. Our approach of using mixed-stage starting material and combining normalized/non-normalized cDNA pools was successful in producing a transcriptome with high gene representation. Based on BLAST searches for known regeneration genes and relevant GO analyses matches, we conclude that our methods captured a significant number of genes that may be involved in regeneration. This transcriptome resource enabled gene expression studies that have provided novel insight into annelid regeneration, yielding the first evidence suggesting that a cell-signaling pathway important in other regenerating systems, Wnt/β-catenin signaling, is initiated during annelid regeneration. Thus, this dataset promises to be instrumental in determining which genes are involved in regeneration processes in *P. leidyi* and will subsequently inform evolution of regeneration studies in the naids as a whole.

The development of genomic resources for the Lophotrochozoa (the large clade of bilaterians including platyhelminths, annelids, and molluscs, among others) has lagged considerably behind that of other major groups of animals. With the advent of less expensive sequencing technologies and an increased appreciation of the value of non-traditional model systems, genomic resources for this group are finally becoming available. Transcriptomes have recently been generated for a range of lophotrochozoan taxa [81-87], including the highly regenerative planarians [88,89]. The growing number of genomic resources for lophotrochozoans promises to help fuel research on a broad range of questions in this large and diverse clade.

## Methods

### Genome size measurement

The genome sizes of five naid species were estimated using the Feulgen image analysis densitometry method [90]. Individuals were obtained from laboratory cultures of the following species: *P. leidyi* (Carolina Biological Supply Company), *Allonais paraguayensis* (Wards Natural Science), *Dero digitata* (originally collected from Edwards Lake, University of Maryland at College Park, USA), *Dero furcata* (Connecticut Valley Biological Supply), and *Paranais litoralis* (originally collected from Herrington Bay, Deale, MD, USA). Fifty or more nuclei were measured from each sample. The Integrated Optical Density of the sample was converted to a genome size value (in picograms) using *Gallus gallus domesticus* (1.25 pg) as a standard.

### Worm culture, sampling, and RNA extraction

To generate material for this sequencing effort, we established twelve replicate lab cultures of a single clonal line of *Pristina leidyi* (PRIle(cbs)cloneA). Each culture was initially started with 100 worms and was maintained at room temperature in 20 cm glass bowls filled with ~1 liter of commercially purchased Poland Spring Water (PSW). To ensure purity of the samples, worm cultures were rinsed frequently to remove algae and debris and cultures were routinely inspected visually for the presence of small metazoans (e.g. rotifers). Worms were fed dried Spirulina powder twice weekly, and water was changed at least 1-2 times per week.

Possible contamination by the dried Spirulina food source was assayed via PCR, using cDNA samples derived from live *Arthrospira platensis* (Spirulina) as a reference. RNA was extracted using TRIReagent (Applied Biosystems), and cDNA was constructed using random oligos and Superscript III reverse transcriptase (Invitrogen). Primers were constructed against the large subunit of *rubisco* (*rbcL*) [GenBank:AY147205.1] and *c-phycocyanin* (*cpc*) [GenBank:AF164139.1] genes of *A. platensis* (Additional file 5).

Worms were collected at a range of stages of regeneration and fission (Figure 1). For the fission material, 1,000 worms that were actively growing and fissioning were collected and starved for 24 hours. To generate regenerating material, 3,485 worms were amputated anteriorly and posteriorly and allowed to regenerate for various lengths of time before collection. Most worms were actively fissioning and consisted of chains of linked zooids at time of amputation. A cut was made 2 body segments anterior to the most anterior fission zone to elicit posterior regeneration. A second cut was made after the 6th body segment of the most posterior zooid to elicit anterior regeneration. If a worm did not consist of at least two nearly formed zooids, a single cut after the 6^th^ body segment was made to elicit anterior regeneration. Because the initiation of regeneration processes holds particular significance for future studies, 1,985 of the regenerating worms in the sample were allowed to regenerate between 0 and 24 hours, which is roughly coincident with the start of blastema formation. Batches of 250 worms were also collected at 1.25 days post-amputation (dpa), 1.75 dpa, 2 dpa, 2.5 dpa, 3 dpa, and 3.5 dpa, when differentiation of adult morphology is nearly complete.

Fissioning and regenerating worms were washed 5x in PSW prior to RNA extraction. RNA was extracted using TRIReagent (Applied Biosystems), and RNA from all samples was then pooled together.

### cDNA library construction

We constructed a pooled cDNA library consisting of a normalized fraction to capture lowly expressed transcripts and a non-normalized fraction to capture large transcripts that might be lost during the PCR amplification steps of the normalization process.

First-strand cDNA (F.S. cDNA) was made using a MINT full-length cDNA synthesis kit (Evrogen) and manufacturer’s instructions. A modified oligo-dT primer with breaks in the homopolymer-T run was used to minimize the negative effects of an extensive homopolymer run on 454 sequence quality (Additional file 5). A portion of the F.S. cDNA was incubated for 2 hours at 15°C with NEB Buffer 2, DNA Polymerase I (New England Biolabs), and RNase H in order to make full-length double-strand cDNA. A fraction of F.S. cDNA was then normalized with Evrogen’s duplex-specific nuclease (DSN). F.S. cDNA-RNA duplexes in hybridization buffer were denatured at 98° for 3 minutes and then allowed to hybridize at 70°C for 5 hours. Preheated DSN at 1/4x concentration was then added and incubated for 20 minutes at 70°C. DSN stop solution was then added, and the sample was incubated for 5 minutes at 70°C. Normalized cDNA was then PCR amplified using an Encyclo PCR kit (Evrogen). PCR conditions were: 1 cycle × 95°C-1 min.; 17 cycles × 95°C-15 sec., 66°C-20 sec., 72°C-3 min.; 1 cycle × 66°C-15 sec., 72°C-3 min. Normalization efficiency was assayed via gel smear and qPCR of genes with known relative abundance (Additional file 5). qPCR analysis was performed using LinRegPCR [91,92].

The non-normalized and normalized cDNA libraries were then pooled in a 1:2 ratio (Figure 1).

### 454 sequencing

Five μg of pooled cDNA library was sent to the Roy J. Carver Biotechnology Center at the University of Illinois for sequencing. The cDNA library was sheared to 500-800 bp in length, 454 sequencing adaptors were ligated onto ends (Additional file 5), and the library was then converted to a single-stranded template library. Three titration runs (each of 1/16 lane) were performed to optimize sequencing conditions. A full plate was then sequenced on a Roche/454 GS FLX Sequencer using Titanium reagents.

### Assembly of 454 sequence reads

Reads from the full plate and three titration runs were assembled using the Newbler Assembler v2.3 (Roche) using default parameters under the cDNA option. Prior to assembly, specified primers and adaptors were trimmed, namely the oligo-dT primer, the MINT PlugOligo adapter and PCR primer (Evrogen), and the 454 sequencing adaptors (Additional file 5).

### Determination of fraction of captured transcripts

The coverage statistic developed by Susko and Roger (2004) estimates the proportion of genes from a cDNA library that is actually represented in the sequence data [39]. Using this method, the unbiased estimate of coverage was calculated for our transcriptome with the equation Ĉ = 1 – *n*_*1*_/*n*, where *n*_*1*_ is the number of singletons in the assembly and *n* is the total number of reads [39,40]. The new gene discovery rate was estimated using the term 1/(1 – Ĉ).

### Annotation and analysis of BLAST hits

The set of 95,644 isotigs was input into the EST2uni annotation pipeline using default parameters, but with PCR marker integration, microarray printing, reciprocal BLAST for orthologues, Gene Ontology, and RFLP integration options turned off [43]. Within EST2uni, the CAP3 assembly parameters were adjusted to *–f 2 –g 100 –p 100 –d 110* to produce an assembly of all singletons, thereby preserving the isotigs produced by Newbler. Isotigs were annotated if they produced BLASTX matches against UniProtKB Release 2010_04 (23-Mar-2010) with E-values less than e^-10^. Parsing of BLAST data for Table 2 was done with custom Perl scripts (available upon request).

Completeness of annotated isotigs in Table 3 was performed using only the largest isotig from each isogroup as a representative for its putative gene locus. A Perl script utilizing BioPerl modules (available upon request) was used for completeness analysis. An isotig was considered complete on either end if it matched within ten amino acids of the corresponding end of the UniProt sequence [40].

The set of max isotigs was also used to identify Gene Ontology (GO) designations using the program Blast2GO [44,45]. Results from BLAST searches against the UniProt database were imported from EST2uni, and GO annotation in Blast2GO was performed with default parameters (E-value threshold of e^-6^).

### Identification of candidate regeneration genes

TBLASTX was used to search the *P. leidyi* isotig dataset for homologs of genes implicated in animal regeneration in the literature. A reciprocal TBLASTX search was then performed against UniProt or the nr database via NCBI to verify the putative identity of candidate regeneration genes in *P. leidyi*.

### Validation of transcript assembly

Transcript validation assays were performed for two isogroups, isogroup08478 (a putative *piwi-like* homolog) and isogroup03233 (a putative *frizzled* homolog). Isotigs of each isogroup were aligned together using ClustalX v2.1 with manual editing by Seaview v4.0 [93,94]. PCR was then performed to verify contigs and isotigs of each isogroup (Additional file 5). PCR amplicons of the expected size were either sequenced directly or cloned into the pGEM-T Easy vector (Promega) prior to sequencing. Sequencing was performed using an Applied Biosystems 3730 × l DNA Analyzer.

Transcript assembly was also verified for four previously known *P. leidyi* genes. BLAST searches against the 454 dataset were performed using the published gene sequences of *Pl-en* [GenBank: AF336055.1], *Pl-otx1* [GenBank: AF336056.1], *Pl-otx2* [GenBank: AF336057.1], and *Pl-nos* [GenBank: GQ369728.1]. GenBank sequences were aligned to transcriptome sequences using Sequencher v.4.7 (Gene Codes Corporation).

### Analysis of gene expression by whole mount in situ hybridization

A ~1250 bp fragment of *Pl-fzA* (isogroup23343) and a ~1300 bp fragment of *Pl-β-cat* (isogroup01340) were amplified by PCR (Additional file 5). Synthesis of sense and antisense riboprobes and *in situ* hybridization were performed as previously described [35].

## Competing interests

The authors declare that they have no competing interests.

## Authors’ contributions

This project was conceived by KGN and AEB. KGN constructed the library, MAC and KGN performed bioinformatic analyses, JLK and KGN empirically assessed assembly quality, KGN characterized gene expression, AF analyzed genome size, and KGN and AEB prepared the manuscript. All authors read and approved the final paper.

## Supplementary Material

Additional file 1 **Genome sizes of five naid species.** Genome sizes of five species of naid worms, including *P. leidyi*, were estimated using the Feulgen image analysis densitometry method. Click here for file

Additional file 2 **PCR assay for metabolic activity in dried Spirulina food.** To assess the possibility of dried Spirulina (used as *P. leidyi* food) contributing to the cDNA library, we used PCR to detect the large subunit of *rubisco* (*rbcL*) and *c-phycocyanin* (*cpc*) of Spirulina (*Arthrospira platensis*). No PCR bands were detectable for either gene in negative water controls (lane 1) while strong bands were detected when cDNA from live Spirulina cultures was used as template (lane 2). Neither Spirulina gene could be detected by PCR in the *P. leidyi* cDNA (lane 3), though PCR of a positive control gene (*Pl-α-tubulin*) produced strong bands using the same template (lane 4). Click here for file

Additional file 3 **Gene Ontology Molecular Function and Cellular Component designations of isotigs.** Representative isotigs were subjected to Gene Ontology (GO) analysis using Blast2GO. Categories are level 2 (A) Molecular Function and (B) Cellular Component designations. Proportion on the y-axis was calculated from the total number of representative isotigs that were annotated with GO terms (11,140). Click here for file

Additional file 4 **Nucleotide alignments for isogroups 08478 and 03233.** Nucleotide alignment of isotigs from (A) isogroup08478, a putative *piwi-like* gene, and (B) isogroup03233, a putative *frizzled* gene. Alignments are diagrammed in Figure 5. Click here for file

Additional file 5 **Primer sequences.** Primer sequences used for cDNA synthesis, 454 adaptors, PCR detection of Spirulina metabolic activity, qPCR of cDNA normalization efficiency, PCR validation of transcript assemblies, and synthesis of *in situ* hybridization probes are provided. All primer sequences are listed 5’→ 3’. Click here for file

## References

[B1] Réaumur RAF Sur les diverses reproductions qui se font dans les Ecrevisse, les Omars, les Crabes, etc. et entr'autres sur celles de leurs Jambes et de leurs Ecailles Mem Acad Royal Sci 1712 223 245

[B2] Trembley A Memoires pour servir a l'Histoire d'un Genre de Polypes d'Eau douce, a Bras en Forme de Cornes 1744

[B3] Bely AE Nyberg KG Evolution of animal regeneration: re-emergence of a field Trends Ecol Evol 2010 25 161 170 19800144 10.1016/j.tree.2009.08.005

[B4] Goss RJ The evolution of regeneration: adaptive or inherent? J Theor Biol 1992 159 241 260 1294847 10.1016/s0022-5193(05)80704-0

[B5] Bely AE Evolutionary loss of animal regeneration: pattern and process Integr Comp Biol 2010 50 4 515 527 21558220 10.1093/icb/icq118

[B6] Sánchez Alvarado A Tsonis PA Bridging the regeneration gap: genetic insights from diverse animal models Nat Rev Genet 2006 7 873 884 17047686 10.1038/nrg1923

[B7] Brockes JP Kumar A Comparative aspects of animal regeneration Annu Rev Cell Dev Biol 2008 24 525 549 18598212 10.1146/annurev.cellbio.24.110707.175336

[B8] Tanaka EM Reddien PW The cellular basis for animal regeneration Dev Cell 2011 21 172 185 21763617 10.1016/j.devcel.2011.06.016 PMC3139400

[B9] Bely AE Distribution of segment regeneration ability in the Annelida Integr Comp Biol 2006 46 508 518 21672762 10.1093/icb/icj051

[B10] Bely AE Sikes JM Latent regeneration abilities persist following recent evolutionary loss in asexual annelids Proc Natl Acad Sci USA 2010 107 1464 1469 19966282 10.1073/pnas.0907931107 PMC2824374

[B11] Scadding SR Phylogenic distribution of limb regeneration capacity in adult Amphibia J Exp Zool 1977 202 57 68

[B12] Scadding SR Limb regeneration in adult amphibia Can J Zool 1981 59 34 46

[B13] Wagner GP Misof BY Evolutionary modification of regenerative capability in vertebrates: a comparative study on teleost pectoral fin regeneration J Exp Zool 1992 261 62 78 1729386 10.1002/jez.1402610108

[B14] Vollrath F Leg regeneration in web spiders and its implications for orb weaver phylogeny Bull Br Arachnol Soc 1990 8 177 184

[B15] Brinkhurst RO Jamieson BGM Aquatic oligochaeta of the world 1971 Edinburgh: Oliver and Boyd

[B16] Zattara EE Bely AE Evolution of a novel developmental trajectory: fission is distinct from regeneration in the annelid Pristina leidyi Evol Dev 2011 13 1 80 95 21210945 10.1111/j.1525-142X.2010.00458.x

[B17] Bely AE Decoupling of fission and regenerative capabilities in an asexual oligochaete Hydrobiologia 1999 406 243 251

[B18] Lengfeld T Watanabe H Simakov O Lindgens D Gee L Law L Schmidt HA Özbek S Bode H Holstein TW Multiple Wnts are involved in Hydra organizer formation and regeneration Dev Biol 2009 330 186 199 19217898 10.1016/j.ydbio.2009.02.004

[B19] Gurley KA Rink JC Sánchez Alvarado A β-catenin defines head versus tail identity during planarian regeneration and homeostasis Science 2008 319 323 327 18063757 10.1126/science.1150029 PMC2755502

[B20] Peterson CP Reddien PW Smed-Bcatenin-1 is required for anteroposterior blastema polarity in planarian regeneration Science 2008 319 327 330 18063755 10.1126/science.1149943

[B21] Adell T Saló E Boutros M Bartscherer K Smed-Evi/Wntless is required for beta-catenin-dependent and -independent processes during planarian regeneration Development 2009 136 905 910 19211673 10.1242/dev.033761

[B22] Almuedo-Castillo M Saló E Adell T Disheveled is essential for neural connectivity and planar cell polarity in planarians Proc Natl Acad Sci USA 2011 108 2813 2818 21282632 10.1073/pnas.1012090108 PMC3041082

[B23] Iglesias M Gomez-Skarmeta JL Saló E Adell T Silencing of Smed-beta-catenin1 generates radial-like hypercephalized planarians Development 2008 135 1215 1221 18287199 10.1242/dev.020289

[B24] Kawakami Y Rodríguez Esteban C Raya M Kawakami H Marti M Dubova I Izpisua Belmonte JC Wnt/beta-catenin signaling regulates vertebrate limb regeneration Genes Dev 2006 20 3232 3237 17114576 10.1101/gad.1475106 PMC1686599

[B25] Lin G Slack JM Requirement for Wnt and FGF signaling in Xenopus tadpole tail regeneration Dev Biol 2008 316 323 335 18329638 10.1016/j.ydbio.2008.01.032

[B26] McClure KD Schubiger G Transdetermination: drosophila imaginal disc cells exhibit stem cell-like potency Int J Biochem Cell Biol 2007 39 1105 1118 17317270 10.1016/j.biocel.2007.01.007 PMC2000801

[B27] Peterson CP Reddien PW A wound-induced Wnt expression program controls planarian regeneration polarity Proc Natl Acad Sci USA 2009 106 17061 17066 19805089 10.1073/pnas.0906823106 PMC2743725

[B28] Schubiger M Sustar A Schubiger G Regeneration and transdetermination: the role of wingless and its regulation Dev Biol 2010 347 315 324 20816798 10.1016/j.ydbio.2010.08.034 PMC2976676

[B29] Stoick-Cooper CL Moon RT Weidinger G Advances in signaling in vertebrate regeneration as a prelude to regenerative medicine Genes Dev 2007 21 11 1292 1315 17545465 10.1101/gad.1540507

[B30] Wall PK Leebens-Mack J Chanderbali AS Barakat A Wolcott E Liang H Landherr L Tomsho LP Hu Y Carlson JE Ma H Schuster SC Soltis DE Soltis PS Altman N dePamphilis CW Comparison of next generation sequencing technologies for transcriptome characterization BMC Genomics 2009 10 347 19646272 10.1186/1471-2164-10-347 PMC2907694

[B31] Margulies M Egholm M Altman WE Attiya S Bader JS Bemben LA Berka J Braverman MS Chen YJ Chen Z Chen Z Dewell SB Du L Fierro JM Gomes XV Godwin BC He W Helgesen S Ho CH Irzyk GP Jando SC Alenquer MLI Jarvie TP Jirage KB Kim JB Knight JR Lanza JR Leamon JH Lefkowitz SM Lei M Li J Lohman KL Lu H Makhijani VB McDade KE McKenna MP Myers EW Nickerson E Nobile JR Plant R Puc BP Ronan MT Roth GT Sarkis GJ Simons JF Simpson JW Srinivasan M Tartaro KR Tomasz A Vogt KA Volkmer GA Wang SH Wang Y Weiner MP Yu P Begley RF Rothberg JM Genome sequencing in microfabricated high-density picolitre reactors Nature 2005 437 376 380 16056220 10.1038/nature03959 PMC1464427

[B32] Rothberg JM Leamon JH The development and impact of 454 sequencing Nat Biotechnol 2008 26 1117 1124 18846085 10.1038/nbt1485

[B33] Gregory TR Hebert PDN Genome size estimates for some oligochaete annelids Can J Zool 2002 80 8 1485 1489

[B34] Gregory TR Animal genome size database 2012 http://www.genomesize.com

[B35] Bely AE Wray GA Evolution of regeneration and fission in annelids: insights from engrailed- and orthodenticle-class gene expression Development 2001 128 2781 2791 11526083 10.1242/dev.128.14.2781

[B36] Zhulidov PA Bogdanova EA Shcheglov AS Vagner LL Khaspekov GL Kozhemyako VB Matz MV Meleshkevitch E Moroz LL Lukyanov SA Shagin DA Simple cDNA normalization using kamchatka crab duplex-specific nuclease Nucleic Acids Res 2004 32 3 e37 14973331 10.1093/nar/gnh031 PMC373426

[B37] Bogdanova EA Shagin DA Lukyanov SA Normalization of full-length enriched cDNA Mol Biosyst 2008 4 205 212 18437263 10.1039/b715110c

[B38] NCBI Short Read Archive http://www.ncbi.nlm.nih.gov/Traces/sra/

[B39] Susko E Roger AJ Estimating and comparing the rates of gene discovery and expressed sequence tag (EST) frequencies in EST surveys Bioinformatics 2004 20 2279 2287 15059814 10.1093/bioinformatics/bth239

[B40] Lee BY Howe AE Conte MA D'Cotta H Pepey E Baroiller JF di Palma F Carleton KL Kocher TD An EST resource for tilapia based on 17 normalized libraries and assembly of 116,899 sequence tags BMC Genomics 2010 11 278 20433739 10.1186/1471-2164-11-278 PMC2874815

[B41] BouillaBase EST databasehttp://cichlid.umd.edu/est2uni//login.php

[B42] Bely lab resourceshttp://www.life.umd.edu/biology/bely/web/belylab/resources.html

[B43] Forment J Gilabert F Robles A Conejero V Nuez F Blanca JM EST2uni: an open, parallel tool for automated EST analysis and database creation, with a data mining web interface and microarray expression data integration BMC Bioinformatics 2008 9 5 18179701 10.1186/1471-2105-9-5 PMC2258287

[B44] Conesa A Gotz S Garcia-Gomez JM Terol J Talon M Robles M Blast2GO: a universal tool for annotation, visualization and analysis in functional genomics research Bioinformatics 2005 21 18 3674 3676 16081474 10.1093/bioinformatics/bti610

[B45] Ashburner M Ball CA Blake JA Botstein D Butler H Cherry JM Davis AP Dolinski K Dwight SS Eppig JT Harris MA Hill DP Issel-Tarver L Kasarskis A Lewis S Matese JC Richardson JE Ringwald M Rubin GM Sherlock G Gene Ontology: tool for the unification of biology Nat Genet 2000 25 1 25 29 10802651 10.1038/75556 PMC3037419

[B46] Campbell LJ Crews CM Wound epidermis formation and function in urodele amphibian limb regeneration Cell Mol Life Sci 2008 65 73 79 18030417 10.1007/s00018-007-7433-z PMC11131783

[B47] Bely AE Sikes JM Acoel and platyhelminth models for stem-cell research J Biol 2010 9 2 14 20236484 10.1186/jbiol223 PMC2871518

[B48] Cho S-J Valles Y Giani VC Seaver EC Weisblat DA Evolutionary dynamics of the wnt gene family: a lophotrochozoan perspective Mol Biol Evol 2010 27 7 1645 1658 20176615 10.1093/molbev/msq052 PMC2912473

[B49] Riddiford N Olson PD Wnt gene loss in flatworms Dev Genes Evol 2011 221 4 187 197 21892738 10.1007/s00427-011-0370-8

[B50] Kato T Miyazaki K Shimizu-Nishikawa K Koshiba K Obara M Mishima HK Yoshizato K Unique expression patterns of matrix metalloproteinases in regenerating newt limbs Dev Dyn 2003 226 2 366 376 12557215 10.1002/dvdy.10247

[B51] Leontovich AA Zhang JS Shimokawa K Nagase H Sarras MP A novel hydra matrix metalloproteinase (HMMP) functions in extracellular matrix degradation, morphogenesis and the maintenance of differentiated cells in the foot process Development 2000 127 4 907 920 10648248 10.1242/dev.127.4.907

[B52] Altincicek B Vilcinskas A Comparative analysis of septic injury-inducible genes in phylogenetically distant model organisms of regeneration and stem cell research, the planarian Schmidtea mediterranea and the cnidarian Hydra vulgaris Frontiers Zool 2008 5 6 10.1186/1742-9994-5-6 PMC2386466 18439314

[B53] Reddien PW Oviedo NJ Jennings JR Jenkin JC Sánchez Alvarado A SMEDWI-2 is a PIWI-like protein that regulates planarian stem cells Science 2005 310 1327 1330 16311336 10.1126/science.1116110

[B54] De Mulder K Pfister D Kuales G Egger B Salvenmoser W Willems M Steger J Fauster K Micura R Borgonie G Ladurner P Stem cells are differentially regulated during development, regeneration, and homeostasis in flatworms Dev Biol 2009 334 198 212 19631639 10.1016/j.ydbio.2009.07.019

[B55] De Mulder K Kuales G Pfister D Willems M Egger B Salvenmoser W Thaler M Gorny A-K Hrouda M Borgonie G Ladurner P Characterization of the stem cell system of the acoel Isodiametra pulchra BMC Dev Biol 2009 9 69 20017953 10.1186/1471-213X-9-69 PMC2806412

[B56] Seipel K Yanze N Schmid V The germ line and somatic stem cell gene Cniwi in the jellyfish Podocoryne carnea Int J Dev Biol 2004 48 1 1 7 15005568 10.1387/ijdb.15005568

[B57] Handberg-Thorsager M Saló E The planarian nanos-like gene Smednos is expressed in germline and eye precursor cells during development and regeneration Dev Genes Evol 2007 217 5 403 411 17390146 10.1007/s00427-007-0146-3

[B58] Wang Y Zayas RM Guo T Newmark PA nanos function is essential for development and regeneration of planarian germ cells Proc Natl Acad Sci USA 2007 104 5901 5906 17376870 10.1073/pnas.0609708104 PMC1851589

[B59] Hayashi T Mizuno N Takada R Takada S Kondoh H Determinative role of Wnt signals in dorsal iris-derived lens regeneration in newt eye Mech Dev 2006 123 11 793 800 17030116 10.1016/j.mod.2006.08.009

[B60] Adell T Marsal M Saló E Planarian GSK3s are involved in neural regeneration Dev Genes Evol 2008 218 2 89 103 18202849 10.1007/s00427-007-0199-3

[B61] D'Jamoos CA McMahon G Tsonis PA Fibroblast growth factor receptors regulate the ability for hindlimb regeneration in Xenopus laevis Wound Repair Regen 1998 6 4 388 397 9824558 10.1046/j.1460-9568.1998.60415.x

[B62] Poss FD Shen JX Nechiporuk A McMahon G Thisse B Thisse C Keating MT Roles for Fgf signaling during zebrafish fin regeneration Dev Biol 2000 222 2 347 358 10837124 10.1006/dbio.2000.9722

[B63] Martin P Parkhurst SM Parallels between tissue repair and embryo morphogenesis Development 2004 131 13 3021 3034 15197160 10.1242/dev.01253

[B64] Tasaki J Shibata N Sakurai T Agata K Umesono Y Role of c-Jun N-terminal kinase activation in blastema formation during planarian regeneration Dev Growth Differ 2011 53 3 389 400 21447099 10.1111/j.1440-169X.2011.01254.x

[B65] Reddien PW Bermange AL Kicza AM Sánchez Alvarado A BMP signaling regulates the dorsal planarian midline and is needed for asymmetric regeneration Development 2007 134 22 4043 4051 17942485 10.1242/dev.007138

[B66] Molina MD Saló E Cebrià F The BMP pathway is essential for re-specification and maintenance of the dorsoventral axis in regenerating and intact planarians Dev Biol 2007 311 1 79 94 17905225 10.1016/j.ydbio.2007.08.019

[B67] Pearl EJ Barker D Day RC Beck CW Identification of genes associated with regenerative success of Xenopus laevis hindlimbs BMC Dev Biol 2008 8 66 18570684 10.1186/1471-213X-8-66 PMC2483965

[B68] Molina MD Neto A Maeso I Luis Gomez-Skarmeta J Saló E Cebrià F Noggin and noggin-like genes control dorsoventral axis regeneration in planarians Curr Biol 2011 21 4 300 305 21295481 10.1016/j.cub.2011.01.016

[B69] Rink JC Gurley KA Elliot SA Sánchez Alvarado A Planarian Hh signaling regulates regeneration polarity and links Hh pathway evolution to cilia Science 2009 326 1406 1410 19933103 10.1126/science.1178712 PMC2861735

[B70] Yazawa S Umesono Y Hayashi T Tarui H Agata K Planarian Hedgehog/Patched establishes anterior-posterior polarity by regulating Wnt signaling Proc Natl Acad Sci USA 2009 106 52 22329 22334 20018728 10.1073/pnas.0907464106 PMC2799762

[B71] Schnapp E Kragl M Rubin L Tanaka EM Hedgehog signaling controls dorsoventral patterning, blastema cell proliferation and cartilage induction during axolotl tail regeneration Development 2005 132 14 3243 3253 15983402 10.1242/dev.01906

[B72] Tsonis PA Vergara MN Spence JR Madhavan M Kramer EL Call MK Santiago WG Vallance JE Robbins DJ Del Rio-Tsonis K A novel role of the hedgehog pathway in lens regeneration Dev Biol 2004 267 2 450 461 15013805 10.1016/j.ydbio.2003.12.005

[B73] Mannini L Deri P Gremigni V Rossi L Salvetti A Batistoni R Two msh/msx-related genes, Djmsh1 and Djmsh2, contribute to the early blastema growth during planarian head regeneration Int J Dev Biol 2008 52 7 943 952 18956324 10.1387/ijdb.072476lm

[B74] Carlson MRJ Bryant SV Gardiner DM Expression of Msx-2 during development, regeneration, and wound healing in axolotl limbs J Exp Zool 1998 282 6 715 723 9846383 10.1002/(sici)1097-010x(19981215)282:6<715::aid-jez7>3.0.co;2-f

[B75] Cho S-J Lee MS Tak ES Lee E Koh KS Ahn CH Park SC Gene expression profile in the anterior regeneration of the earthworm using expressed sequence tags Biosci Biotechnol Biochem 2009 73 1 29 34 19129665 10.1271/bbb.80391

[B76] Mullen LM Bryant SV Torok MA Blumberg B Gardiner DM Nerve dependency of regeneration: the role of Distal-less and FGF signaling in amphibian limb regeneration Development 1996 122 11 3487 3497 8951064 10.1242/dev.122.11.3487

[B77] Juliano CE Swartz SZ Wessel GM A conserved germline multipotency program Development 2010 137 24 4113 4126 21098563 10.1242/dev.047969 PMC2990204

[B78] Neira-Oviedo M Tsyganov-Bodounov A Lycett GJ Kokoza V Raikhel AS Krzywinski J The RNA-Seq approach to studying the expression of mosquito mitochondrial genes Insect Mol Biol 2011 20 2 141 152 20958808 10.1111/j.1365-2583.2010.01053.x

[B79] Wilhelm BT Landry J-R RNA-Seq-quantitative measurement of expression through massively parallel RNA-sequencing Methods 2009 48 3 249 257 19336255 10.1016/j.ymeth.2009.03.016

[B80] Wang Z Gerstein M Snyder M RNA-Seq: a revolutionary tool for transcriptomics Nat Rev Genet 2009 10 1 57 63 19015660 10.1038/nrg2484 PMC2949280

[B81] Henry JQ Perry KJ Fukui L Alvi N Differential localization of mRNAs during early development in the mollusc, Crepidula fornicata Integr Comp Biol 2010 50 5 720 733 21558235 10.1093/icb/icq088

[B82] Lambert JD Chan XY Spiecker B Sweet HC Characterizing the embryonic transcriptome of the snail Ilyanassa Integr Comp Biol 2010 50 5 768 777 21558239 10.1093/icb/icq121

[B83] Clark MS Thorne MAS Vieira FA Cardoso JCR Power DM Peck LS Insights into shell deposition in the Antarctic bivalve Laternula elliptica: gene discovery in the mantle transcriptome using 454 pyrosequencing BMC Genomics 2010 11 362 20529341 10.1186/1471-2164-11-362 PMC2896379

[B84] Hou R Bao Z Wang S Su H Li Y Du H Hu J Wang S Hu X Transcriptome sequencing and de novo analysis for Yesso scallop (Patinopecten yessoensis) using 454 GS FLX PLoS One 2011 6 6 e21560 21720557 10.1371/journal.pone.0021560 PMC3123371

[B85] Gong P Pirooznia M Guan X Perkins EJ Design, validation and annotation of transcriptome-wide oligonucleotide probes for the oligochaete annelid Eisenia fetida PLoS One 2010 5 12 e14266 21170345 10.1371/journal.pone.0014266 PMC2999564

[B86] Heyland A Vue Z Voolstra CR Medina M Moroz LL Developmental transcriptome of Aplysia californica J Exp Zool B 2011 316B 2 113 134 10.1002/jez.b.21383 PMC4028319 21328528

[B87] Milan M Coppe A Reinhardt R Cancela LM Leite RB Saavedra C Ciofi C Chelazzi G Patarnello T Bortoluzzi S Bargelloni L Transcriptome sequencing and microarray development for the Manila clam, Ruditapes philippinarum: genomic tools for environmental monitoring BMC Genomics 2011 12 234 21569398 10.1186/1471-2164-12-234 PMC3107815

[B88] Abril JF Cebrià F Rodríguez Esteban G Horn T Fraguas S Calvo B Bartscherer K Saló E Smed454 dataset: unraveling the transcriptome of Schmidtea mediterranea BMC Genomics 2010 11 731 21194483 10.1186/1471-2164-11-731 PMC3022928

[B89] Adamidi C Wang Y Gruen D Mastrobuoni G You X Tolle D Dodt M Mackowiak SD Gogol-Doering A Oenal P Rybak A Ross E Sánchez Alvarado A Kempa S Dieterich C Rajewsky N Chen W De novo assembly and validation of planaria transcriptome by massive parallel sequencing and shotgun proteomics Genome Res 2011 21 1193 1200 21536722 10.1101/gr.113779.110 PMC3129261

[B90] Hardie DC Gregory TR Hebert PDN From pixels to picograms: a beginners' guide to genome quantification by Feulgen image analysis densitometry J Histochem Cytochem 2002 50 6 735 749 12019291 10.1177/002215540205000601

[B91] Ramakers C Ruijter JM Deprez RHL Moorman AFM Assumption-free analysis of quantitative real-time polymerase chain reaction (PCR) data Neurosci Lett 2003 339 1 62 66 12618301 10.1016/s0304-3940(02)01423-4

[B92] Ruijter JM Ramakers C Hoogaars WMH Karlen Y Bakker O van den Hoff MJB Moorman AFM Amplification efficiency: linking baseline and bias in the analysis of quantitative PCR data Nucleic Acids Res 2009 37 6 e45 19237396 10.1093/nar/gkp045 PMC2665230

[B93] Gouy M Guindon S Gascuel O SeaView version 4: a multiplatform graphical user interface for sequence alignment and phylogenetic tree building Mol Biol Evol 2010 27 2 221 224 19854763 10.1093/molbev/msp259

[B94] Larkin MA Blackshields G Brown NP Chenna R McGettigan PA McWilliam H Valentin F Wallace IM Wilm A Lopez R Thompson JD Gibson TJ Higgins DG Clustal W and clustal X version 2.0 Bioinformatics 2007 23 21 2947 2948 17846036 10.1093/bioinformatics/btm404

