# Supplementary material for: Transcriptome characterization via 454 pyrosequencing of the annelid Pristina leidyi, an emerging model for studying the evolution of regeneration
Source: BMC Genomics. 2012 Jun 29;13:287. doi: 10.1186/1471-2164-13-287 (PMC3464666; doi:10.1186/1471-2164-13-287)
Supplement: Additional file 1 — Genome sizes of five naid species. Genome sizes of five species of naid worms, including P. leidyi, were estimated using the Feulgen image analysis densitometry method. [file 1471-2164-13-287-S1.pdf]

Additional File 1 – Genome sizes of five naid species

Naidine species	C-value (pg)
Pristina leidyi	1.37
Allonais paraguayensis	0.69
Dero digitata	0.54
Dero furcata	0.77
Paranais litoralis	1.09
