# Supplementary figures and images for: Transcriptome characterization via 454 pyrosequencing of the annelid Pristina leidyi, an emerging model for studying the evolution of regeneration
Source: BMC Genomics. 2012 Jun 29;13:287. doi: 10.1186/1471-2164-13-287 (PMC3464666; doi:10.1186/1471-2164-13-287)

**Additional File 2 – PCR assay for metabolic activity in dried Spirulina food**

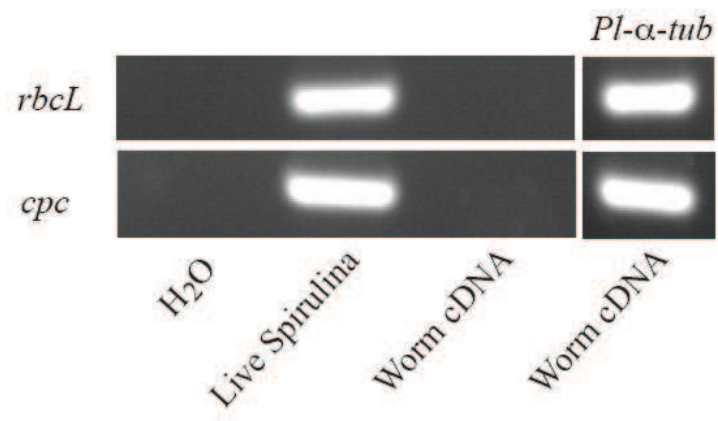

Supplement: Additional file 2 — PCR assay for metabolic activity in dried Spirulina food. To assess the possibility of dried Spirulina (used as P. leidyi food) contributing to the cDNA library, we used PCR to detect the large subunit of rubisco (rbcL) and c-phycocyanin (cpc) of Spirulina (Arthrospira platensis). No PCR bands were detectable for either gene in negative water controls (lane 1) while strong bands were detected when cDNA from live Spirulina cultures was used as template (lane 2). Neither Spirulina gene could be detected by PCR in the P. leidyi cDNA (lane 3), though PCR of a positive control gene (Pl-α-tubulin) produced strong bands using the same template (lane 4). [file 1471-2164-13-287-S2.pdf]
