# Supplementary material for: Transcriptome characterization via 454 pyrosequencing of the annelid Pristina leidyi, an emerging model for studying the evolution of regeneration
Source: BMC Genomics. 2012 Jun 29;13:287. doi: 10.1186/1471-2164-13-287 (PMC3464666; doi:10.1186/1471-2164-13-287)

Additional File 3 – Gene Ontology Molecular Function and Cellular Component designations of isotigs

A

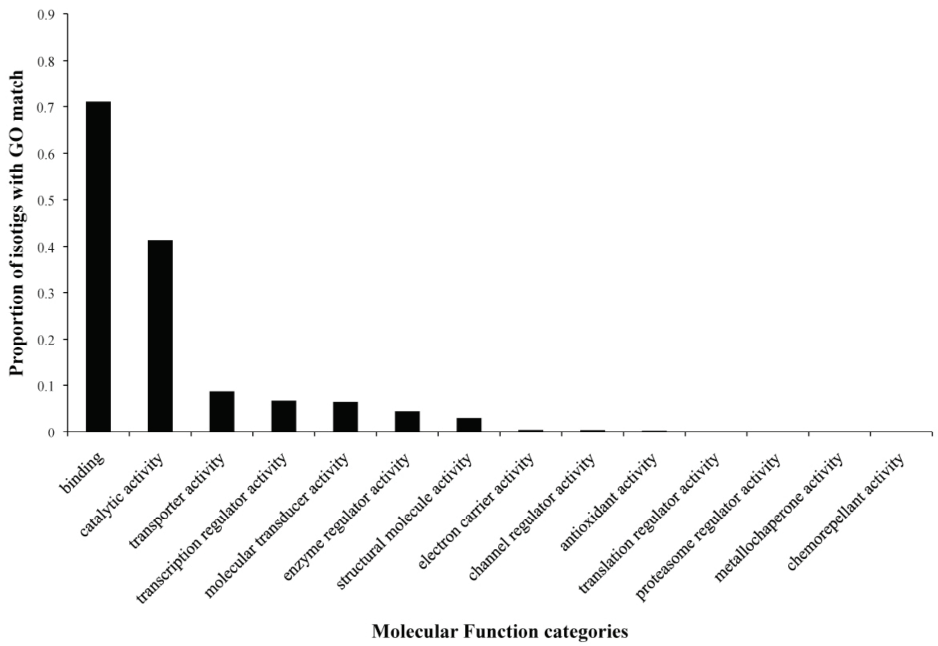

B

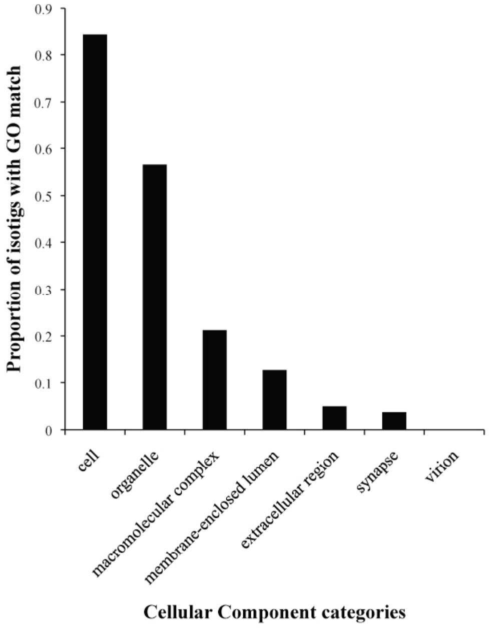

Supplement: Additional file 3 — Gene Ontology Molecular Function and Cellular Component designations of isotigs. Representative isotigs were subjected to Gene Ontology (GO) analysis using Blast2GO. Categories are level 2 (A) Molecular Function and (B) Cellular Component designations. Proportion on the y-axis was calculated from the total number of representative isotigs that were annotated with GO terms (11,140). [file 1471-2164-13-287-S3.pdf]
