# Supplementary material for: Transcriptome characterization via 454 pyrosequencing of the annelid Pristina leidyi, an emerging model for studying the evolution of regeneration
Source: BMC Genomics. 2012 Jun 29;13:287. doi: 10.1186/1471-2164-13-287 (PMC3464666; doi:10.1186/1471-2164-13-287)
Supplement: Additional file 4 — Nucleotide alignments for isogroups 08478 and 03233. Nucleotide alignment of isotigs from (A) isogroup08478, a putative piwi-like gene, and (B) isogroup03233, a putative frizzled gene. Alignments are diagrammed in Figure 5. [file 1471-2164-13-287-S4.pdf]

Additional File 4 – Nucleotide alignments for isogroups 08478 and 03233

A Isogroup08478 – putative *piwi-like* gene

```
isotig33900      ATAATCCAGGCAAATCAACGATCATACACTTACAACCAGGACACGGCTGATTGGTCGAAA
isotig33901      ATAATCCAGGCAAATCAACGATCATACACTTACAACCAGGACACGGCTGATTGGTCGAAA
                 ************************************************************

isotig33900      GAGATGAGAGGACTACCCCTGCTGAACCCAGCCCATCTAACCAACTGGGTACTCATCTAC
isotig33901      GAGATGAGAGGACTACCCCTGCTGAACCCAGCCCATCTAACCAACTGGGTACTCATCTAC
                 ************************************************************

isotig33900      ACGAGTCGAGATGCTGGCAATGCAAGTGACCTGAGTTCAACACTAGTGAAAGTCTGTCAG
isotig33901      ACGAGTCGAGATGCTGGCAATGCAAGTGACCTGAGTTCAACACTAGTGAAAGTCTGTCAG
                 ************************************************************

isotig33900      CCAATGGGAATTCGTGTCGACCAACCACGAACATGCCAGTTGCCTGATGATAGTACAAAC
isotig33901      CCAATGGGAATTCGTGTCGACCAACCACGAACATGCCAGTTGCCTGATGATAGTACAAAC
                 ************************************************************

isotig33900      ACATTCGTACGAGCACTGAAGGAATCTATTGACGACACGGTTTGCATGGTGGTGATAATA
isotig33901      ACATTCGTACGAGCACTGAAGGAATCTATTGACGACACGGTTTGCATGGTGGTGATAATA
                 ************************************************************

isotig33900      CTACCCACAAACAGGAAGGATCGTTATGATGCCATCAAAACCATCTGCTGTGTTGAGCAG
isotig33901      CTACCCACAAACAGGAAGGATCGTTATGATGCCATCAAAACCATCTGCTGTGTTGAGCAG
                 ************************************************************

isotig33900      CCAGTTCCAAGCCAAGTTGTATTGGCTCGTACTTTATCCAAGAAGCAGATGTTGATGTCT
isotig33901      CCAGTTCCAAGCCAAGTTGTATTGGCTCGTACTTTATCCAAGAAGCAGATGTTGATGTCT
                 ************************************************************

isotig33900      GTGGCAACCAAAATTGCCATTCAATTGAACGTCAAAATGGGCGGCGAAGTATGGGCGCTG
isotig33901      GTGGCAACCAAAATTGCCATTCAATTGAACGTCAAAATGGGCGGCGAAGTATGGGCGCTG
                 ************************************************************

isotig33900      GATATACCTATCAAGAATGTCATGATTATTGGAATTGATACTTATCACGATTCTGCCAAG
isotig33901      GATATACCTATCAAGAATGTCATGATTATTGGAATTGATACTTATCACGATTCTGCCAAG
                 ************************************************************

isotig33900      AAAGGTCGATCTGTTGGAGCATTCATTGCCAGCATGAACTCAACCTTTACACGTTATTAT
isotig33901      AAAGGTCGATCTGTTGGAGCATTCATTGCCAGCATGAACTCAACCTTTACACGTTATTAT
                 ************************************************************

isotig33900      TCACGATGTGCATTCCAATCAAGTCACGAGGAACTACAGAATGCTCTGAAAATATGTATG
isotig33901      TCACGATGTGCATTCCAATCAAGTCACGAGGAACTACAGAATGCTCTGAAAATATGTATG
                 ************************************************************

isotig33900      CAGAGTGCTTTGAAGAATTACCATGCCGTTAATGGAGTTCTGCCTGAGAAAGTCATAGTC
isotig33901      CAGAGTGCTTTGAAGAATTACCATGCCGTTAATGGAGTTCTGCCTGAGAAAGTCATAGTC
                 ************************************************************

isotig33900      TTCCGTGATGGAGTTGGGGATGGACAGCTGGCAACTGTTCACGAGCATGAAGTTCGTCAG
isotig33901      TTCCGTGATGGAGTTGGGGATGGACAGCTGGCAACTGTTCACGAGCATGAAGTTCGTCAG
                 ************************************************************

isotig33900      CTGACTGACTGCTTTCAGGCACTTGGACCAGATTACAGTCCAAAGTTTGCAGTAATCGTG
isotig33901      CTGACTGACTGCTTTCAGGCACTTGGACCAGATTACAGTCCAAAGTTTGCAGTAATCGTG
                 ************************************************************

isotig33900      GTCAAGAAGCGCATCAACTCCCGTTTCTTCATGACAGAGAGGGGCCAAACTACAAATCCA
isotig33901      GTCAAGAAGCGCATCAACTCCCGTTTCTTCATGACAGAGAGGGGCCAAACTACAAATCCA
                 ************************************************************

isotig33900      CGTGCAGGAACCATCATTGACACAGAGGCAACGCGGCCAGAATGGTACGATTTCTTCTTG
isotig33901      CGTGCAGGAACCATC---------------------------------------------
                 ***************

isotig33900      GTGAGTCAGTCGGTTCGTCAGGGAACGGTTGCTCCAACTCACTACAATGTCATCTACGAT
isotig33901      ------------------------------------------------------------


isotig33900      ACAACTGGTTTCAAGCCGGATCACATGCAACGTTTGGCTTACAAACTGTGCCATTTGTAC
isotig33901      ------------------------------------------------------------
```

```
isotig33900     TACAACTGGCAGGGAACGATTCGTGTGCCGGCGCCTTGCCAGTATGCTCACAAAATGGCA
isotig33901     ------------------------------------------------------------

isotig33900     TTCCTGGTTGGTCAGTCTCTTCATAGAGATCCATCTCTGCGCCTAGCCGACAAGCTGTTC
isotig33901     -----------------------------------TCTGCGCCTAGCCGACAAGCTGTTC
                                                   *************************

isotig33900     TTCCTGTAGTATCTCTGACTCGGCTGCTGAACTGTTACACTGTCGATGCTGACGTGCTGA
isotig33901     TTCCTGTAGTATCTCTGACTCGGCTGCTGAACTGTTACACTGTCGATGCTGACGTGCTGA
                ************************************************************

isotig33900     ATATCAAGATTTTATTTCATCTCAGTTAGCAGTTGATTCTGCGTTGTTGTGATAATTGAT
isotig33901     ATATCAAGATTTTATTTCATCTCAGTTAGCAGTTGATTCTGCGTTGTTGTGATAATTGAT
                ************************************************************

isotig33900     CTACTTTGATGATTAATACTTTTTTTCAATGTGTTCATTCGTAATACAGTGGACTGTAGT
isotig33901     CTACTTTGATGATTAATACTTTTTTTCAATGTGTTCATTCGTAATACAGTGGACTGTAGT
                ************************************************************

isotig33900     TTTTTAATGTACCATTGGATTTGTATGGTTTCACGGAGAAGATGCTCAGCTTTTCTTGCC
isotig33901     TTTTTAATGTACCATTGGATTTGTATGGTTTCACGGAGAAGATGCTCAGCTTTTCTTGCC
                ************************************************************

isotig33900     GTTTGAGGAAGTGTTTCGGAATGATGGTGTCGGCCATTACAAGAGGCTGACATTCACATC
isotig33901     GTTTGAGGAAGTGTTTCGGAATGATGGTGTCGGCCATTACAAGAGGCTGACATTCACATC
                ************************************************************

isotig33900     TATTGTATTTTTAGCTAAGTCGTGCCGAGTTGTTTGGACTGTTGCAATACACGTTGTCTT
isotig33901     TATTGTATTTTTAGCTAAGTCGTGCCGAGTTGTTTGGACTGTTGCAATACACGTTGTCTT
                ************************************************************

isotig33900     GAATGCACCTATGTGTATTTTCTCAAGTATTGAAGCCATCGGAAAGGAACTTTGATCGAG
isotig33901     GAATGCACCTATGTGTATTTTCTCAAGTATTGAAGCCATCGGAAAGGAACTTTGATCGAG
                ************************************************************

isotig33900     TTCACGTTTGATGTCTGAATATTTTTCTACTAGCTGTTCCGACGTTAGTGTAGTTTTGTT
isotig33901     TTCACGTTTGATGTCTGAATATTTTTCTACTAGCTGTTCCGACGTTAGTGTAGTTTTGTT
                ************************************************************

isotig33900     GATACGTTAACATCCATTTCGTCAGTATTGTTGATAACGCATCTCTAGTGCGACCACATT
isotig33901     GATACGTTAACATCCATTTCGTCAGTATTGTTGATAACGCATCTCTAGTGCGACCACATT
                ************************************************************

isotig33900     GCTGCAGTAGCACGTGCGTGCTCGGCCTGCATTTTTTTGACAATTTGTTTGCGATAGATT
isotig33901     GCTGCAGTAGCACGTGCGTGCTCGGCCTGCATTTTTTTGACAATTTGTTTGCGATAGATT
                ************************************************************

isotig33900     CTGTTTGAGATTTGTGAAGTAGCGGATGTTATGCATAAATAATTCTGCATCCTGATGCAT
isotig33901     CTGTTTGAGATTTGTGAAGTAGCGGATGTTATGCATAAATAATTCTGCATCCTGATGCAT
                ************************************************************

isotig33900     AATTGTTCTTGGTGGTGCTGATTTAAGAGATGCCCAACTGATTTAAGAGATGCCCAACTG
isotig33901     AATTGTTCTTGGTGGTGCTGATTTAAGAGATGCCCAACTGATTTAAGAGATGCCCAACTG
                ************************************************************

isotig33900     TTGAAACTATTTCATCCTGTTTATACCGGGCATTAAAACTGCATTTACGTCTGCATACGA
isotig33901     TTGAAACTATTTCATCCTGTTTATACCGGGCATTAAAACTGCATTTACGTCTGCATACGA
                ************************************************************

isotig33900     TGAATTGATGATTGTTTTGGAGTTTGAAAAA
isotig33901     TGAATTGATGATTGTTTTGGAGTTTGAAAAA
                *******************************
```

B Isogroup03233 – putative *frizzled* gene

```
isotig19223      ------------------------------------------------------------
isotig19224      CCCGTAGTCGACGCGTCTGCAACATGTCCAGCGTTTTCCAACTGACGACAACTGTTGCAG
isotig19225      ------------------------------------------------------------
isotig19226      ------------------------------------------------------------
isotig19227      CCCGTAGTCGACGCGTCTGCAACATGTCCAGCGTTTTCCAACTGACGACAACTGTTGCAG
isotig19228      ------------------------------------------------------------

isotig19223      ------------------------------------------------------------
isotig19224      ATGTCAACGTTGATGATTTGACGTTTTCGTACGACAGCAACACTTGTCGCAGTCGTTCAT
isotig19225      ------------------------------------------------------------
isotig19226      ------------------------------------------------------------
isotig19227      ATGTCAACGTTGATGATTTGACGTTTTCGTACGACAGCAACACTTGTCGCAGTCGTTCAT
isotig19228      ------------------------------------------------------------

isotig19223      ------------------------------------------------------------
isotig19224      GTCGACATGGCTGCATGTTGCCGTATTGATCGTAAACTGCTATCTTAACCGACCGAGCGA
isotig19225      ------------------------------------------------------------
isotig19226      ------------------------------------------------------------
isotig19227      GTCGACATGGCTGCATGTTGCCGTATTGATCGTAAACTGCTATCTTAACCGACCGAGCGA
isotig19228      ------------------------------------------------------------

isotig19223      ------------------------------------------------------------
isotig19224      TATTCGGCGCGAAGTGCCTTTGCTCGATTTTATGTCGACGAGCTGCGTTTGACCGATTTT
isotig19225      ------------------------------------------------------------
isotig19226      ------------------------------------------------------------
isotig19227      TATTCGGCGCGAAGTGCCTTTGCTCGATTTTATGTCGACGAGCTGCGTTTGACCGATTTT
isotig19228      ------------------------------------------------------------

isotig19223      ----------------------CCGAACTGTTTCAAGTTTTATCGCTGTCGCTACAAACG
isotig19224      TGTGCGGGTTGTGTTCCGAAA----AACTGTTTCAAGTTTTATCGCTGTCGCTACAAACG
isotig19225      ------------------------------------------------------------
isotig19226      ----------------------CCGAACTGTTTCAAGTTTTATCGCTGTCGCTACAAACG
isotig19227      TGTGCGGGTTGTGTTCCGAAA----AACTGTTTCAAGTTTTATCGCTGTCGCTACAAACG
isotig19228      ------------------------------------------------------------

isotig19223      TAGGCTACGCACGCACGGTCTGGCGGGCGTTCGCTTGGAGCGTCGCTCGCCTTCGAAACA
isotig19224      TAGGCTACGCACGCACGGTCTGGCGGGCGTTCGCTTGGAGCGTCGCTCGCCTTCGAAACA
isotig19225      ------------------------------------------------------------
isotig19226      TAGGCTACGCACGCACGGTCTGGCGGGCGTTCGCTTGGAGCGTCGCTCGCCTTCGAAACA
isotig19227      TAGGCTACGCACGCACGGTCTGGCGGGCGTTCGCTTGGAGCGTCGCTCGCCTTCGAAACA
isotig19228      ------------------------------------------------------------

isotig19223      TCGAGATAACACATACGTCACACAAAGATAGCAGCGAAATAATGCGGTCGTCGTCGTCGT
isotig19224      TCGAGATAACACATACGTCACACAAAGATAGCAGCGAAATAATGCGGTCGTCGTCGTCGT
isotig19225      ------------------------------------------------------------
isotig19226      TCGAGATAACACATACGTCACACAAAGATAGCAGCGAAATAATGCGGTCGTCGTCGTCGT
isotig19227      TCGAGATAACACATACGTCACACAAAGATAGCAGCGAAATAATGCGGTCGTCGTCGTCGT
isotig19228      ------------------------------------------------------------

isotig19223      -GATCAGCTGGAATCCGTGTACTTGCGCTGCTTCTTAATTCCACTGCCATCCTGCAGTAT
isotig19224      -GATCAGCTGGAATCCGTGTACTTGCGCTGCTTCTTAATTCCACTGCCATCCTGCAGTAT
isotig19225      ------------------------------------------------------------
isotig19226      ------------------------------------------------------------
isotig19227      ------------------------------------------------------------
isotig19228      ------------------------------------------------------------

isotig19223      CAGCGGACTTGAGTCAATATGGAATCGAGTTATGCAGCACCAATCGGACGTTTGGCAGCC
isotig19224      CAGCGGACTTGAGTCAATATGGAATCGAGTTATGCAGCACCAATCGGACGTTTGGCAGCC
isotig19225      ------------------------------------------------------------
isotig19226      ------------------------------------------------------------
isotig19227      ------------------------------------------------------------
isotig19228      ------------------------------------------------------------
```

```
isotig19223      AGCGCTCTTTTGCTTCAGCTGTTACTGACCGGCAGTCACCAGATGCCGTCCGCTGTCGGT
isotig19224      AGCGCTCTTTTGCTTCAGCTGTTACTGACCGGCAGTCACCAGATGCCGTCCGCTGTCGGT
isotig19225      ------------------------------------------------------------
isotig19226      ------------------------------------------------------------
isotig19227      ------------------------------------------------------------
isotig19228      ------------------------------------------------------------

isotig19223      TTCGGTTCGCAGCAACAGCAACAGCAGCAACAACAGCCGCAGCAACAGCAGCAGCATTCG
isotig19224      TTCGGTTCGCAGCAACAGCAACAGCAGCAACAACAGCCGCAGCAACAGCAGCAGCATTCG
isotig19225      ------------------------------------------------------------
isotig19226      ------------------------------------------------------------
isotig19227      ------------------------------------------------------------
isotig19228      ------------------------------------------------------------

isotig19223      ATGATGTCGCACCATCACCTGC--------------------------------------
isotig19224      ATGATGTCGCACCATCACCTGC--------------------------------------
isotig19225      -----------------------CGTTCCTCGTGCTGTATTTCTTCGGCATGGCTGGATC
isotig19226      ------------------------------------------------------------
isotig19227      ------------------------------------------------------------
isotig19228      -----------------------CGTTCCTCGTGCTGTATTTCTTCGGCATGGCTGGATC

isotig19223      ------------------------------------------------------------
isotig19224      ------------------------------------------------------------
isotig19225      GCTCTGGTGGGTCGCTCTGACGGTCGCATTTTACTTGTCGGCCGGACGGAAGTGGAGTCG
isotig19226      ------------------------------------------------------------
isotig19227      ------------------------------------------------------------
isotig19228      GCTCTGGTGGGTCGCTCTGACGGTCGCATTTTACTTGTCGGCCGGACGGAAGTGGAGTCG

isotig19223      ------------------------------------------------------------
isotig19224      ------------------------------------------------------------
isotig19225      AGAAGCGATCGAAGCGCGGTCGGCCTACTTTCACGTCGCCGGCTGGACACTTCCGGCCGT
isotig19226      ------------------------------------------------------------
isotig19227      ------------------------------------------------------------
isotig19228      AGAAGCGATCGAAGCGCGGTCGGCCTACTTTCACGTCGCCGGCTGGACACTTCCGGCCGT

isotig19223      ------------------------------------------------------------
isotig19224      ------------------------------------------------------------
isotig19225      GAAAACGATCGCCATATTGGCGCTTCATCGTGTCGACGCGGACGAATTGACGGGACTCTG
isotig19226      ------------------------------------------------------------
isotig19227      ------------------------------------------------------------
isotig19228      GAAAACGATCGCCATATTGGCGCTTCATCGTGTCGACGCGGACGAATTGACGGGACTCTG

isotig19223      ------------------------------------------------------------
isotig19224      ------------------------------------------------------------
isotig19225      TTACGTCGGCAACGTCGACCGAGACGCGCTGGTCGCCTTCGTTGTCGTTCCGCTGTTGGC
isotig19226      ------------------------------------------------------------
isotig19227      ------------------------------------------------------------
isotig19228      TTACGTCGGCAACGTCGACCGAGACGCGCTGGTCGCCTTCGTTGTCGTTCCGCTGTTGGC

isotig19223      ------------------------------------------------------------
isotig19224      ------------------------------------------------------------
isotig19225      CTATCTCATCGTCGGCATAGCGTTTATCGCTGCAGGTTTCACTGCTATGTTCCACATCCG
isotig19226      ------------------------------------------------------------
isotig19227      ------------------------------------------------------------
isotig19228      CTATCTCATCGTCGGCATAGCGTTTATCGCTGCAGGTTTCACTGCTATGTTCCACATCCG

isotig19223      ------------------------------------------------------------
isotig19224      ------------------------------------------------------------
isotig19225      CAAAGACCTTCTGCGTGGCGGCAGCGGCGAGAACGACGACGGAAACATAATCGCTCCGGG
isotig19226      ------------------------------------------------------------
isotig19227      ------------------------------------------------------------
isotig19228      CAAAGACCTTCTGCGTGGCGGCAGCGGCGAGAACGACGACGGAAACATAATCGCTCCGGG

isotig19223      ------------------------------------------------------------
isotig19224      ------------------------------------------------------------
isotig19225      AGGAACGCAAACTATTCGGAAGTTGGAAAACTAATGGCAAAAATCGGCGTGTTCTCTGTG
isotig19226      ------------------------------------------------------------
isotig19227      ------------------------------------------------------------
isotig19228      AGGAACGCAAACTATTCGGAAGTTGGAAAACTAATGGCAAAAATCGGCGTGTTCTCTGTG
```

```
isotig19223      ------------------------------------------------------------
isotig19224      ------------------------------------------------------------
isotig19225      CTCTATACCGTACCGGCGACGTGTGTCGTTGCGTGTTTCCTGTACCGTCGCATGAACATG
isotig19226      ------------------------------------------------------------
isotig19227      ------------------------------------------------------------
isotig19228      CTCTATACCGTACCGGCGACGTGTGTCGTTGCGTGTTTCCTGTACCGTCGCATGAACATG

isotig19223      ------------------------------------------------------------
isotig19224      ------------------------------------------------------------
isotig19225      GACATCTGGCGGCAGCGAGCTGCCGCTGGCTTGGACTGTGGCGGCCCTCGTAAGCATCTG
isotig19226      ------------------------------------------------------------
isotig19227      ------------------------------------------------------------
isotig19228      GACATCTGGCGGCAGCGAGCTGCCGCTGGCTTGGACTGTGGCGGCCCTCGTAAGCATCTG

isotig19223      ------------------------------------------------------------
isotig19224      ------------------------------------------------------------
isotig19225      AGCTTAGTAGGGTCTGAAGACGAACCGGACTGCGAGCCTCTGAGGCAATCGATACCGCCG
isotig19226      ------------------------------------------------------------
isotig19227      ------------------------------------------------------------
isotig19228      AGCTTAGTAGGGTCTGAAGACGAACCGGACTGCGAGCCTCTGAGGCAATCGATACCGCCG

isotig19223      ------------------------------------------------------------
isotig19224      ------------------------------------------------------------
isotig19225      ATAGAGGTCGAGATGCTGAGGTTGTTCATGTTATTGGCGGGCGGAATGACCAGCTTCGTG
isotig19226      ------------------------------------------------------------
isotig19227      ------------------------------------------------------------
isotig19228      ATAGAGGTCGAGATGCTGAGGTTGTTCATGTTATTGGCGGGCGGAATGACCAGCTTCGTG

isotig19223      ------------------------------------------------------------
isotig19224      ------------------------------------------------------------
isotig19225      TGGATCTGCTCGAGGAAAACTATTTTGACGTGGTACCGATTTTTGACGCGAAGTGGAGGA
isotig19226      ------------------------------------------------------------
isotig19227      ------------------------------------------------------------
isotig19228      TGGATCTGCTCGAGGAAAACTATTTTGACGTGGTACCGATTTTTGACGCGAAGTGGAGGA

isotig19223      ------------------------------------------------------------
isotig19224      ------------------------------------------------------------
isotig19225      CGAATTGGGTCCCGACATCTGTCGCCAGGCGCCAGTGCTCACCAAAACAGACCGGTGCGA
isotig19226      ------------------------------------------------------------
isotig19227      ------------------------------------------------------------
isotig19228      CGAATTGGGTCCCGACATCTGTCGCCAGGCGCCAGTGCTCACCAAAACAGACCGGTGCGA

isotig19223      ------------------------------------------------------------
isotig19224      ------------------------------------------------------------
isotig19225      TCAGATGCGGCAGCGCTACCGTCGGCGGTGGGGGATTGCGTTTAGCTACGCCAGCCACCG
isotig19226      ------------------------------------------------------------
isotig19227      ------------------------------------------------------------
isotig19228      TCAGATGCGGCAGCGCTACCGTCGGCGGTGGGGGATTGCGTTTAGCTACGCCAGCCACCG

isotig19223      ------------------------------------------------------------
isotig19224      ------------------------------------------------------------
isotig19225      GATCACGTGATATTCACACCGCGGCTGC-TTCAGCAGCCGTCGTCGTCGTCGT----CTG
isotig19226      ---------------------------------------------------------CTG
isotig19227      ---------------------------------------------------------CTG
isotig19228      GATCACGTGATATTCACACCGCGGCTGC--------------------------------

isotig19223      ------------------------------------------------------------
isotig19224      ------------------------------------------------------------
isotig19225      CAGCTGGAAAATGTGAACGGATCACGATACCTCTATGTAAGGATATGAAATACAATATGA
isotig19226      CAGCTGGAAAATGTGAACGGATCACGATACCTCTATGTAAGGATATGAAATACAATATGA
isotig19227      CAGCTGGAAAATGTGAACGGATCACGATACCTCTATGTAAGGATATGAAATACAATATGA
isotig19228      ------------------------------------------------------------
```

```
isotig19223      ------------------------------------------------------------
isotig19224      ------------------------------------------------------------
isotig19225      CGCGGATGCCGAACCTGGTCGGTCAAACTAATCAAAAGGACGCAGCGCTTCAGGTGCACG
isotig19226      CGCGGATGCCGAACCTGGTCGGTCAAACTAATCAAAAGGACGCAGCGCTTCAGGTGCACG
isotig19227      CGCGGATGCCGAACCTGGTCGGTCAAACTAATCAAAAGGACGCAGCGCTTCAGGTGCACG
isotig19228      ------------------------------------------------------------

isotig19223      ------------------------------------------------------------
isotig19224      ------------------------------------------------------------
isotig19225      AGTCCATACCGCTGATACAGTTCGGTTGTTCAAACCTGTTGAAGTTTTTCCTCTGTTCTT
isotig19226      AGTCCATACCGCTGATACAGTTCGGTTGTTCAAACCTGTTGAAGTTTTTCCTCTGTTCTT
isotig19227      AGTCCATACCGCTGATACAGTTCGGTTGTTCAAACCTGTTGAAGTTTTTCCTCTGTTCTT
isotig19228      ------------------------------------------------------------

isotig19223      -------------------------------CTACCGGAAGTGCGACACGTCTTATCCTG
isotig19224      -------------------------------CTACCGGAAGTGCGACACGTCTTATCCTG
isotig19225      TGTACGCACCAATGTGTACCGAACAAGTCG------------------------------
isotig19226      TGTACGCACCAATGTGTACCGAACAAGTCG------------------------------
isotig19227      TGTACGCACCAATGTGTACCGAACAAGTCG------------------------------
isotig19228      -------------------------------CTACCGGAAGTGCGACACGTCTTA-CCTG

isotig19223      CATTCGAACAGTATCACGACTCGAATTTGATTGGTGTATTTATGTATTTTGTCCCTATAA
isotig19224      CATTCGAACAGTATCACGACTCGAATTTGATTGGTGTATTTATGTATTTTGTCCCTATAA
isotig19225      ------------------------------------------------------------
isotig19226      ------------------------------------------------------------
isotig19227      ------------------------------------------------------------
isotig19228      CATTCGAACAGTATCACGACTCGAATTTGATTGGTGTATTTATGTATTTTGTCCCTATAA

isotig19223      TTGTACGTAGCACGGGATTGATCATGGAGTTGTTACGG
isotig19224      TTGTACGTAGCACGGGATTGATCATGGAGTTGTTACGG
isotig19225      --------------------------------------
isotig19226      --------------------------------------
isotig19227      --------------------------------------
isotig19228      TTGTACGTAGCACGGGATTGATCATGGAGTTGTTACGG
```
