# Supplementary material for: Transcriptome characterization via 454 pyrosequencing of the annelid Pristina leidyi, an emerging model for studying the evolution of regeneration
Source: BMC Genomics. 2012 Jun 29;13:287. doi: 10.1186/1471-2164-13-287 (PMC3464666; doi:10.1186/1471-2164-13-287)
Supplement: Additional file 5 — Primer sequences. Primer sequences used for cDNA synthesis, 454 adaptors, PCR detection of Spirulina metabolic activity, qPCR of cDNA normalization efficiency, PCR validation of transcript assemblies, and synthesis of in situ hybridization probes are provided. All primer sequences are listed 5’→ 3’. [file 1471-2164-13-287-S5.pdf]

**Additional File 5 – Primer sequences**

**A. Primers for cDNA library construction (trimmed from raw sequences prior to sequence assembly)**

modified oligo-dT reverse transcription primer:

AAGCAGTGGTATCAACGCAGAGTACTTTTGTTTTTTTTTCTTT
TTTTTTTVN

MINT 5' PlugOligo adaptor:

AAGCAGTGGTATCAACGCAGAGTACGGGGG-P

MINT M1 PCR primer:

AAGCAGTGGTATCAACGCAGAGT

**B. 454 sequencing adaptors (trimmed from raw sequences prior to sequence assembly)**

| | |
|---|---|
| 454 A1 | CCATCTCATCCCTGCGTGTCTCCGACTCAG |
| 454 B1 | CCTATCCCCTGTGTGCCTTGGCAGTCTCAG |

**C. Primers for PCR assay of metabolic activity in dried Spirulina food (Additional File 2)**

| | |
|---|---|
| rbcL-A+ | AACCTCCATCGTGGGTAACG |
| rbcL-C- | CAGCGGTGAAGAAGTCGTGC |
| cpc-D+ | AGTCGCCTAGGAATCAGAGC |
| cpc-E- | GTTGGGTAGCAAAACCACGG |
| atub-A+ | ACCCACACCACTCTGGAGCG |
| atub-B- | TGATTTCAGCAACGGTGAGC |

**D. Primers for qPCR assays of normalization efficiency (Figure 2)**

| | |
|---|---|
| PRIle-βactin-Q1+ | ACGCCAACACCGTCATGTCT |
| PRIle-βactin-Q2- | GATCCAGACGGAGTATTTGC |
| PRIle-αtub-Q1+ | CTTACACCAACCTGAACCGT |
| PRIle-αtub-Q2- | CTGGAACTCGGTCAGATCGA |
| PRIle-wnt1Q1+ | TCGGTGACGTCATCAAAGAC |
| PRIle-wnt1Q2- | AGGCTGACGATGGTGAGAGT |
| PRIle-otx2Q1+ | CGGTATCCGGACATATTCAC |
| PRIle-otx2Q2- | GGAACTGACCTGCTCAACTG |
| PRIle-hoxZ(ST42)-Q1+ | TCGAAATCGCTGCCTCGCTA |
| PRIle-hoxZ(ST42)-Q2- | GGCACCCGATGAAGATCCAT |

**E. Primers for validation of contigs and isotigs (Figure 5)**

Isogroup08478 (*piwi-like*):

| | |
|---|---|
| Piwi_contigA+ | ATAATCCAGGCAAATCAACGATC |
| Piwi_contigA- | GATGGTTCCTGCACGTGGATTTG |
| Piwi_contigB+ | ATTGACACAGAGGCAACGCGG |
| Piwi_contigB- | GATGGATCTCTATGAAGAGACTG |
| Piwi_contigC+ | TCTGCGCCTAGCCGACAAGCT |
| Piwi_contigC- | CTCCAAAACAATCATCAATTCATCG |

Isogroup03233 (*frizzled*):

| | |
|---|---|
| Fz_contigA+ | CCCGTAGTCGACGCGTCTGC |
| Fz_contigA- | TTTCGGAACACAACCCGCACA |
| Fz_contigA2+ | TGTCCAGCGTTTTCCAACTGAC |
| Fz_contigA2- | GTCAAACGCAGCTCGTCGAC |
| Fz_contigB+ | CTGTTTCAAGTTTTATCGCTGTC |
| Fz_contigB- | ACGACGACGACGACCGCATT |
| Fz_contigB2+ | TAGGCTACGCACGCACGGTC |
| Fz_contigB2- | TTCGCTGCTATCTTTGTGTGAC |
| Fz_contigC+ | GATCAGCTGGAATCCGTGTACT |
| Fz_contigC- | GCAGGTGATGGTGCGACATC |
| Fz_contigC2+ | CGCTGCTTCTTAATTCCACTG |
| Fz_contigC2- | CGAATGCTGCTGCTGTTGCTG |
| Fz_contigD+ | CGTTCCTCGTGCTGTATTTCTTC |
| Fz_contigD- | GCAGCCGCGGTGTGAATATCAC |
| Fz_contigD2+ | TTCGGCATGGCTGGATCGCT |
| Fz_contigD2- | GTGGCTGGCGTAGCTAAACG |
| Fz_contigE+ | CTGCAGCTGGAAAATGTGAACG |
| Fz_contigE- | CGACTTGTTCGGTACACATTG |
| Fz_contigE2+ | GATCACGATACCTCTATGTAAGG |
| Fz_contigE2- | TGGTGCGTACAAAGAACAGAGGA |
| Fz_contigF+ | CTACCGGAAGTGCGACACGTC |
| Fz_contigF- | CCGTAACAACTCCATGATCAATC |

**F. Primers for *in situ* hybridization probe synthesis (Figure 7)**

*Pl-fzA* (isogroup23343):

| | |
|---|---|
| Pl-fzA-A+ | CGTTCCTCGTGCTGTATTTCTTC |
| Pl-fzA-B- | TAAGATTCCGAAGTGATTCGG |

*Pl-β-cat* (isogroup01340):

| | |
|---|---|
| Pl-β-cat-Q+ | GTAGATTCAGGAATTCATTC |
| Pl-β-cat-T- | GTAAGGCACATATCGTTGGC |
